# LINE-1 Mediated Insertion into *Poc1a* (Protein of Centriole 1 A) Causes Growth Insufficiency and Male Infertility in Mice

**DOI:** 10.1371/journal.pgen.1005569

**Published:** 2015-10-23

**Authors:** Krista A. Geister, Michelle L. Brinkmeier, Leonard Y. Cheung, Jennifer Wendt, Melissa J. Oatley, Daniel L. Burgess, Kenneth M. Kozloff, James D. Cavalcoli, Jon M. Oatley, Sally A. Camper

**Affiliations:** 1 Graduate Program in Cellular and Molecular Biology, University of Michigan, Ann Arbor, Michigan, United States of America; 2 Department of Human Genetics, University of Michigan, Ann Arbor, Michigan, United States of America; 3 Roche NimbleGen, Inc., Research and Development, Madison, Wisconsin, United States of America; 4 School of Molecular Biosciences and Center for Reproductive Biology, Washington State University, Pullman, Washington, United States of America; 5 Department of Orthopedic Surgery, University of Michigan, Ann Arbor, Michigan, United States of America; 6 Department of Computational Medicine and Bioinformatics, University of Michigan, Ann Arbor, Michigan, United States of America; University of Pennsylvania, UNITED STATES

## Abstract

Skeletal dysplasias are a common, genetically heterogeneous cause of short stature that can result from disruptions in many cellular processes. We report the identification of the lesion responsible for skeletal dysplasia and male infertility in the spontaneous, recessive mouse mutant *chagun*. We determined that *Poc1a*, encoding protein of the centriole 1a, is disrupted by the insertion of a processed *Cenpw* cDNA, which is flanked by target site duplications, suggestive of a LINE-1 retrotransposon-mediated event. Mutant fibroblasts have impaired cilia formation and multipolar spindles. Male infertility is caused by defective spermatogenesis early in meiosis and progressive germ cell loss. Spermatogonial stem cell transplantation studies revealed that *Poc1a* is essential for normal function of both Sertoli cells and germ cells. The proliferative zone of the growth plate is small and disorganized because chondrocytes fail to re-align after cell division and undergo increased apoptosis. *Poc1a* and several other genes associated with centrosome function can affect the skeleton and lead to skeletal dysplasias and primordial dwarfisms. This mouse mutant reveals how centrosome dysfunction contributes to defects in skeletal growth and male infertility.

## Author Summary

Severe short stature in humans has many causes including defects in skeletal and hormonal growth regulation. Primordial dwarfisms are congenital growth defects that involve mutations in genes for DNA repair, DNA replication, splicing of U12 introns, and centrosome dynamics. We discovered that the spontaneous, dwarf mouse mutant *chagun* is caused by loss-of-function of the gene *Poc1a*, which encodes protein of the centriole 1A. Mutants exhibit disproportionate dwarfism, and bones formed by endochondral and intramembranous ossification processes are affected. The epiphyseal growth plates of their long bones are disorganized. The *chagun* males are infertile due to Sertoli cell dysfunction and the failure of germ cells to complete meiosis. The *chagun* mouse is a model for human dwarfism and provides insight into the mechanism whereby this centriolar protein affects bone growth and spermatogenesis.

## Introduction

Normal adult stature in humans is achieved primarily through regulation of long bone growth, which occurs through endochondral ossification [1,2]. This process begins with the differentiation of mesenchymal stem cells into chondrocytes in regions of the body where skeletal elements will eventually reside. Hypertrophic differentiation of these chondrocytes directs the vascularization of the forming element, allowing osteoblasts to enter and commence mineralization of the cartilage-based template. Pools of cells at the epiphyses of the long bones retain their cartilage identity as a means to secure the progressive addition of bone matrix throughout the period of skeletal growth. These structures are the epiphyseal growth plates. Tight control of chondrocyte proliferation and terminal hypertrophic differentiation allows new bone tissue to replace terminally differentiated chondrocytes in a spatially and temporally regulated manner, ensuring the proper growth of the skeletal elements and the individual overall. Disruption of these processes can lead to skeletal dysplasias.

The growth plate maintains a highly ordered architecture to carry out its function and is divided into three distinct zones: the resting, the proliferative, and the hypertrophic zone [1,2]. Perturbation of growth plate organization can result in profound growth defects in mice and humans [3–5]. Resting chondrocytes have a low rate of cell proliferation, and the plane of cell division is not controlled. In contrast, rapidly dividing chondrocytes in the proliferative zone undergo directed cytokinesis orthogonal to the main direction of bone growth, and then intercalate to form distinctive columns of disc-shaped chondrocytes [3,6]. Major orchestrators of growth plate architecture include the primary cilium [3], the WNT-planar cell polarity [6], TGFβ, BMP, and hedgehog signaling pathways [4].

Primordial dwarfisms are a subset of growth insufficiency disorders that are classified as forms of skeletal dysplasia [7,8]. Primordial dwarfism typically involves a proportionate reduction in longitudinal growth that commences early in fetal life [7]. Primordial dwarfisms result in profound reductions in height, and many are associated with head and/or facial dysmorphisms [1]. The primordial dwarfisms include Seckel Syndrome, Microcephalic Osteodysplastic Primordial Dwarfism I-III (MOPD I-III), and Meier-Gorlin Syndrome (MGS) [7]. These disorders interfere with processes that are intimately connected to the cell cycle rather than an endocrine disturbance. Common pathways that are disrupted in primordial dwarfism patients include: DNA repair [9–12], DNA replication [13–16], centrosome dynamics [12–15,17–23], and splicing of minor, or U12 introns, in mRNA [24,25].

We report discovery of the gene responsible for autosomal recessive skeletal dysplasia and male infertility in *chagun* mice [26]. The mutation is a LINE-1 mediated insertion of a processed *Cenpw* cDNA into *Poc1a*, which encodes protein of the centriole 1A. This disruption causes exon skipping, and the mutant protein lacks a highly conserved WD-40 domain necessary for normal spindle pole and cilia formation. POC1A is necessary for normal growth plate architecture, craniofacial development, and spermatogenesis. The mechanism underlying the growth defect is cell death and failure to regulate polarized cell division and intercalation at the growth plate. This animal model clarifies the molecular basis for the clinical features in human patients with *POC1A* mutations and predicts that male patients will be azoospermic [21,22].

## Results

### *chagun* causes disproportionate growth of the skull and other skeletal elements

**Fig 1:**
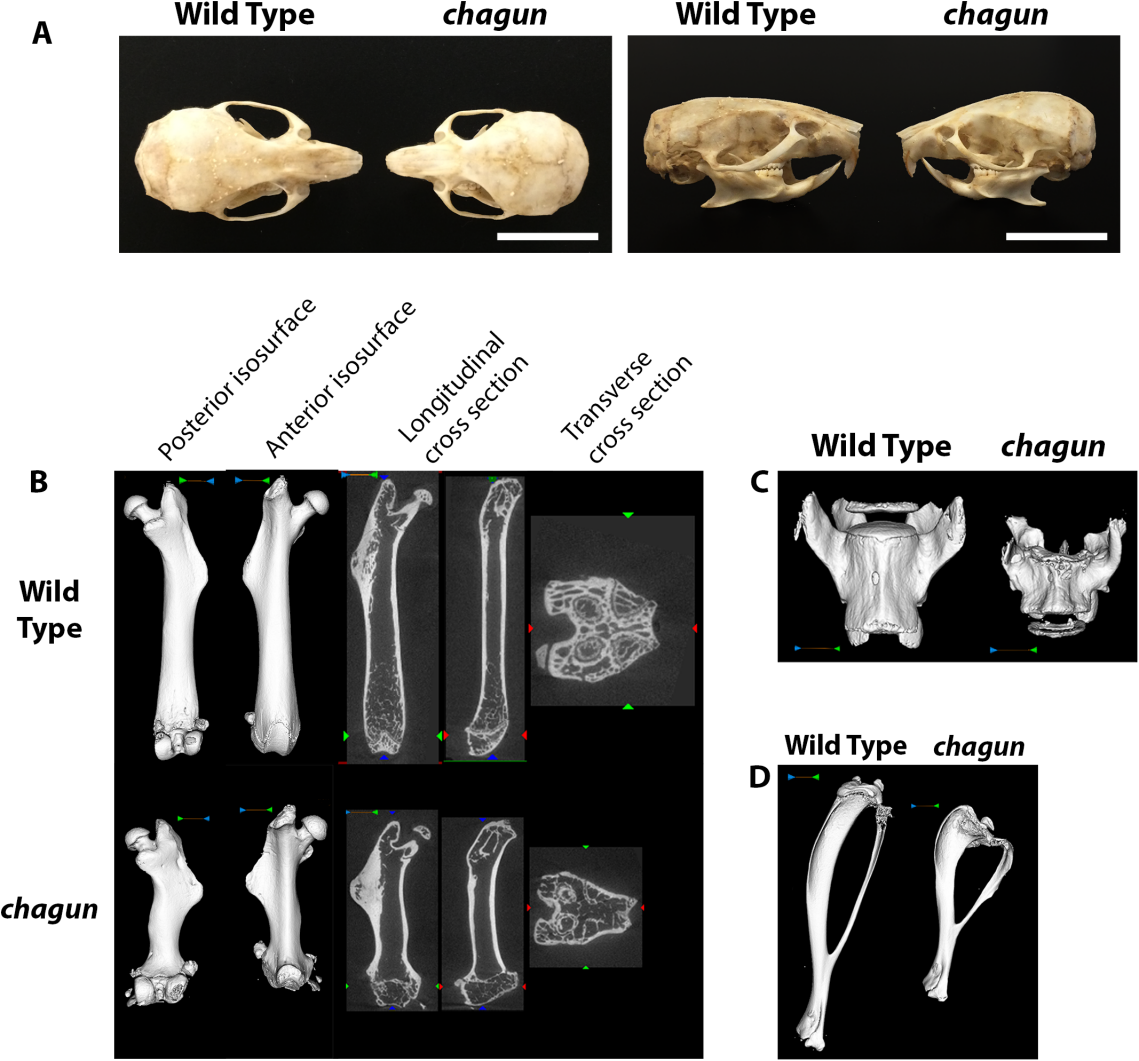
Abnormal skull and long bone morphology in *chagun* mutants. A. Skulls of wild type and *chagun* mice were stripped of soft tissues by incubation in a dermastid beetle colony. Scale bar = 1cm. B-D. Micro-CT scans and volume reconstructions of the femur (B), lumbar vertebrae (C), and tibia (D) in 4-month-old male wild type and *chagun* mice.

The *chagun* mutation arose on the DBA/2J strain and was mapped to a 6 Mb region of Chr 9 using recombinant progeny from an F1 x F1 intercross with CAST/EiJ [26]. Initial studies revealed an autosomal recessive disproportionate growth disorder, likely caused by disorganization of the proliferative zone of the growth plate. We used dermestid beetle preparations to characterize the mutant bones (Fig 1). The *chagun* mutant skull has a slightly domed and disproportionate shape. The skull and mandible are foreshortened along the length, but the widths are similar to wild type. Incisor and molar teeth appear normal.

We used micro-CT to assess bone architecture and mineralization (Fig 1). Representative cross sections and 3D volumetric renderings of 4-month-old male mice were selected based on medial bone mineral content and are shown with matched display settings. We found no differences in cortical mineralization, but the trabecular bone was thinner in both vertebrae and metaphyseal long bones of the mutants. The *chagun* femora are obviously short, with undulating cortical bone and lateral third trochanter irregularities. Disorganization at the distal femoral growth plate is apparent, likely leading to the noticeable lack of trabecular bone in the metaphysis. Metaphyseal periosteal shaping appears to be significantly impacted by the mutation, with abnormal anterior-posterior and medial-lateral shaping. Similar findings were observed in the tibia, with decreased tibial length, proximal tibial growth plate abnormalities, and excessive proximal tibial and mid-fibular flaring. Skeletal shortening extends to the axial skeleton, with pronounced loss of height in lumbar vertebrae.

### The *chagun* growth defect is caused by a mutation in *Poc1a*

Genome-wide exome capture and high throughput sequencing of a *chagun* genomic DNA sample was conducted (http://www.broadinstitute.org/mouse-mutant-resequencing). No obvious deleterious mutations were uncovered in the mapped critical region using this approach. There were some synonymous changes and a single missense mutation that encoded an amino acid present in normal individuals of other species. Some variants were detected in intronic regions captured with the exons, but none appeared likely to disrupt splicing or create ectopic splice donors or acceptors (S1 Table).

We extended our search using regional DNA capture to enrich for all non-repetitive genomic DNA in the most broadly defined 8.5 Mb *chagun* critical region (*D9Mit183*-*D9Mit212*). We captured genomic DNA from an obligate carrier (*cha/+*) and a known mutant (*cha/cha*), and obtained high throughput sequence with at least 20X coverage for approximately 90% of the bases sequenced from both samples. Manual sequence curation was carried out to detect insertions, deletions, inversions, etc. An insertion of an L1Md-G_F_-5 end non-LTR transposable element [27] was detected in an intron of the gene that encodes collagen type VI, alpha 6 (*Col6a6*). This element is not reported in 16 mouse reference genomes (http://www.sanger.ac.uk/resources/mouse/genomes/) [28]. PCR amplification and Sanger sequencing of this region in DNA samples from affected, unaffected, and DBA/2J mice indicates that it is present in all three samples. Thus, this transposable element does not cause the mutant phenotype. The difficulty inherent in sequencing and mapping repetitive elements likely led to omission of this element from the reference genomes.

**Fig 2:**
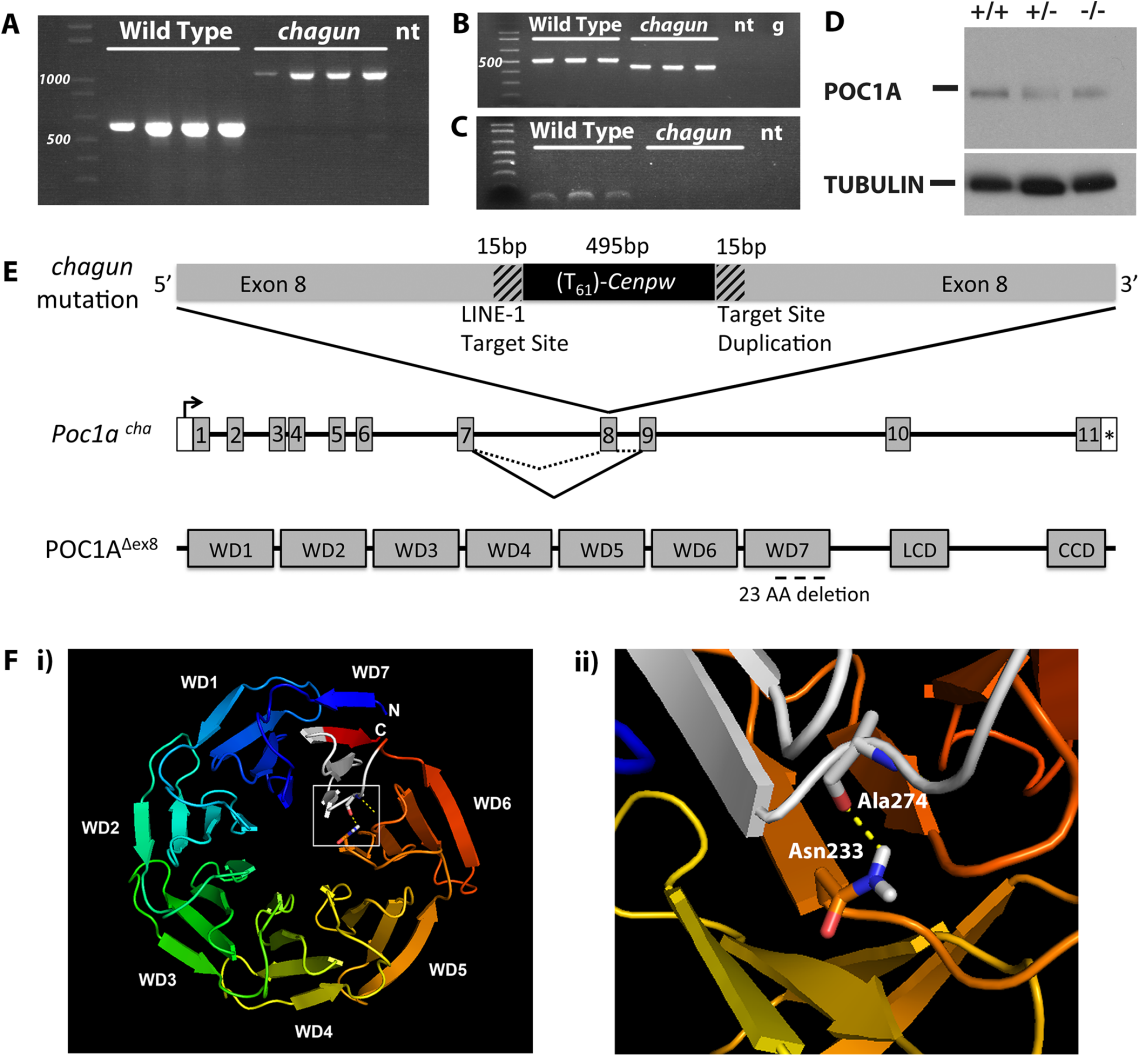
Insertion of a processed cDNA into exon 8 of *Poc1a* underlies the *chagun* phenotype. A. Normal and *chagun* mutant genomic DNA samples (N = 4 mice per genotype) were amplified with primers flanking *Poc1a* exon 8. The products were separated by gel electrophoresis and stained with ethidium bromide. A 1,100 bp product was produced from the *chagun* genomic DNA samples and the predicted product 613 bp in wild-type genomic DNA. B. *Poc1a* transcripts were analyzed by RT-PCR using RNA from tibia (N = 3 per genotype) and primers specific to exons 5 and 10. A 526 bp product was detected in wild types, but a ~450 bp product was detected in *chagun* mutants. C. *Poc1a* transcripts in tibia were analyzed using primers in exons 7 and 8. The expected size product was detected in wild types but no amplification products were detected in *chagun* mutants. D. Protein extracts from P3 tibiae of wild type (+/+), heterozygote (+/-) and *chagun* mutants (-/-) were run on a 12% SDS-PAGE gel, transferred to a membrane, and probed with primary antibodies specific for POC1A and tubulin. Both images are exposures taken from the same blot. E. Schematic diagram of the processed *Cenpw* cDNA insertion into *Poc1a*, the effects on mRNA splicing, and the predicted protein. A portion of the *Poc1a* coding sequence is duplicated (hatched regions) at the 3’ end of the *Cenpw* insertion (black) within exon 8. The insertion results in skipping exon 8, and the predicted protein lacks a portion of the last WD-40 repeat domain. F. Molecular modeling of WD40 domains and amino acid interactions. i) Molecular model of the WD domains of mouse POC1A. The N- and C-termini of POC1A, which do not form part of the WD domains, have not been modeled. The *chagun* mutation is indicated in white within WD7. ii) A predicted hotspot of amino acid interactions, Asn233, forms a hydrogen bond with Ala274, which resides within WD7 and the *chagun* deletion. LCD: Low complexity domain, CCD: Coiled-coil domain, no template (nt), Genomic DNA (g).

Manual sequence curation revealed a steep decrease in sequence coverage near exon 8 of *Poc1a*, which encodes protein of the centriole 1A (S1 Fig). This is more pronounced in the mutant than the heterozygote. In addition, many captured fragments from this region have mismatched paired end sequencing reads, where one read maps to chromosome 9 and the other to chromosomes 5, 10, or 12. PCR amplification of *Poc1a* exon 8 and flanking intronic regions produced a product that is ~500 bp larger in genomic DNA from *chagun* mice than in unaffected animals (Fig 2). Sanger sequencing of the genomic amplification products indicated that exon 8 is disrupted by the insertion of a nearly full-length transcript of centromere protein W (*Cenpw*) including a 61 nucleotide poly-A tail that is 24 bp downstream from the common polyadenylation sequence AUUAAA (S1 Fig). The total size of the insertion is 495 bp, and it includes a 5’ untranslated region (UTR) lacking only seven bp relative to the published transcription start site, all three exons, and a 3’UTR. This insertion is unique to *chagun*; it is not present in any of the 16 additional strains analyzed. The bona fide *Cenpw* gene is located on mouse chromosome 10, and processed *Cenpw* pseudogenes exist on chromosomes 5 and 12. The sequence of the *Cenpw* insert in *Poc1a* shares 100% identity with the exons of *Cenpw* on chromosome 10, and 90% and 84% identity with the pseudogenes on chromosomes 12 and 5, respectively. Thus, the inserted sequences of *Cenpw* in exon 8 of *Poc1a* are derived from a processed transcript of the bona fide *Cenpw* gene.

The insertion does not delete any DNA from *Poc1a* exon 8. The exon is interrupted by the insertion of the *Cenpw* transcript in reverse orientation relative to *Poc1a*. The point of insertion is flanked by short (15 bp) stretches of identical *Poc1a* sequence (5’-CACCGTTGCC**TTTTC**-3’). This arrangement is consistent with target-site duplications characteristic of LINE-1 retrotransposon-mediated insertions (Reviewed in [29]), and with the consensus LINE-1 endonuclease ORF2 insertion site sequence (5’ TTTTC|A), just before the insertion of the *Cenpw* transcript [30–33].

### The processed *Cenpw* cDNA insertion causes exon skipping but retains the *Poc1a* reading frame

RNA extracted from tibiae was prepared for RT-PCR analysis of *Poc1a* transcripts. The *chagun* cDNA produced a smaller amplification product than that detected in normal tibia (Fig 2). Sanger sequencing of the mutant RT-PCR product revealed that exon 8 of *Poc1a* is skipped precisely. No splicing into exon 8 was detected in *chagun* mutants (Fig 2). Quantitative RT-PCR was carried out using probes designed to amplify exons 2–3 at the 5’ end of the *Poc1a* transcript and exon 11–12 at the 3’ end of the transcript. The same level of transcripts were detected in RNA from wild type and *chagun* mutant tibiae using probes for both the 5’ or 3’ ends of the transcript (S1 Fig). This suggests that exon 8 is cleanly skipped and that the mutant transcript is similar in stability to the wild type.

Skipping exon 8 preserves the reading frame of the *Poc1a* transcript. The mutant transcript is predicted to produce a POC1A protein that lacks 23 amino acids within the seventh and final 40 amino acid WD40 repeat (Fig 2). Western blots were carried out to detect POC1A protein in tibiae using a polyclonal antibody generated against full-length human POC1A, which is highly conserved (89% identity, UniProt) between human and mouse. A single band of similar intensity was detected in wild type and mutant tibia (Fig 2). Pan-tubulin immunoreactivity was used to normalize protein preparations. The molecular structure of mouse POC1A was modeled using the WD40 structure predictor algorithm [34]. Molecular modeling suggests that the deletion would destabilize the seven bladed propeller structure characteristic of WD40 repeat domain proteins because the first and seventh WD40 repeats normally interlock to form the propeller, and the mutant protein lacks 23 of 40 amino acids that would comprise the seventh propeller. Additionally, the WDSP algorithm predicts certain amino acids to be hotspots for protein-protein interactions based on their biochemical properties and their location on the top face of the propeller structure [35]. There is a predicted hotspot in WD6, Asn233, which normally forms hydrogen bonds with Ala274, a residue that is deleted in *chagun* mutants. The loss of this interaction likely disrupts the protein-protein interactions of Asn233.

### The *chagun* mutant is rescued by a *Poc1a* BAC transgene and phenocopied by *Poc1a* null mice

**Fig 3:**
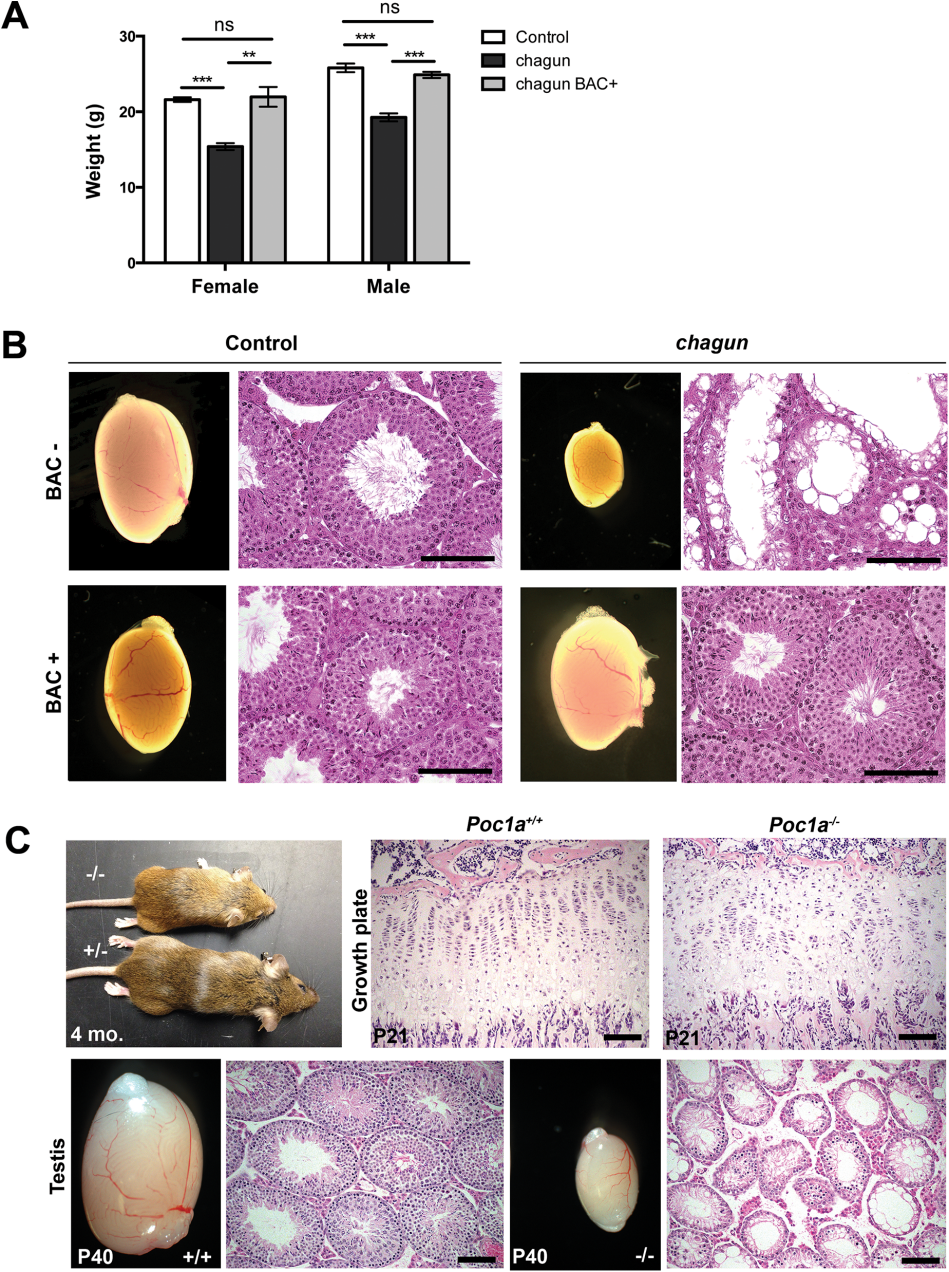
A *Poc1a* BAC transgene rescues *chagun*, and a null allele phenocopies *chagun*. A. Female and male mice of the indicated genotypes were weighed at 6 wks of age. N = 5-19/group. B. Adult male animals of the indicated genotypes were analyzed for testis morphology (0.8X objective) and histology. Testis sections were stained with hematoxylin and eosin. Scale bar = 100 μm. C. Bones were collected from *Poc1a* ^+/+^ and *Poc1a* ^-/-^ animals at P21 and testes from P40 animals. Samples were fixed, sectioned and stained with hematoxylin and eosin. Scale bar = 100 μm.

To validate the causal role of the mutation in *Poc1a* in the *chagun* phenotype, we undertook a BAC transgenic rescue experiment [36]. The selected BAC clone RP24-384G5 contains four genes in addition to *Poc1a*: *Twf2*, *Tlr9*, *Alas1*, and *Dusp7*. These genes do not cause problems when overexpressed, nor do their known loss-of-function phenotypes mimic any aspect of *chagun* mutants (S2 Table). Two of three independent BAC transgene insertion sites in founder mice provided completely penetrant correction of the *chagun* phenotype and caused no abnormalities in control transgene-positive animals. Transgenic *chagun* animals exhibit normal growth and testicular development (Fig 3). Body weight and length, as well as testicular weight, appearance, and histology were normal. This evidence supports our assertion that loss of *Poc1a* function due to the exon 8 insertion causes the *chagun* mutant phenotype.

We obtained embryonic stem (ES) cells from the knockout mouse project in which exons 3–6 of the *Poc1a* locus were deleted and replaced by a gene trap internal ribosome entry site (IRES) LacZ and a neomycin resistance expression cassette (S2 Fig). We used these ES cells to generate mice with a null allele of *Poc1a* [37]. Heterozygous carriers for the LacZ knock-in were normal, and mice homozygous for this null allele phenocopied the growth insufficiency and hypogonadism of *chagun* mutants (Fig 3).

### POC1A is normally expressed in tibial growth plates and seminiferous tubules of wild type mice

**Fig 4:**
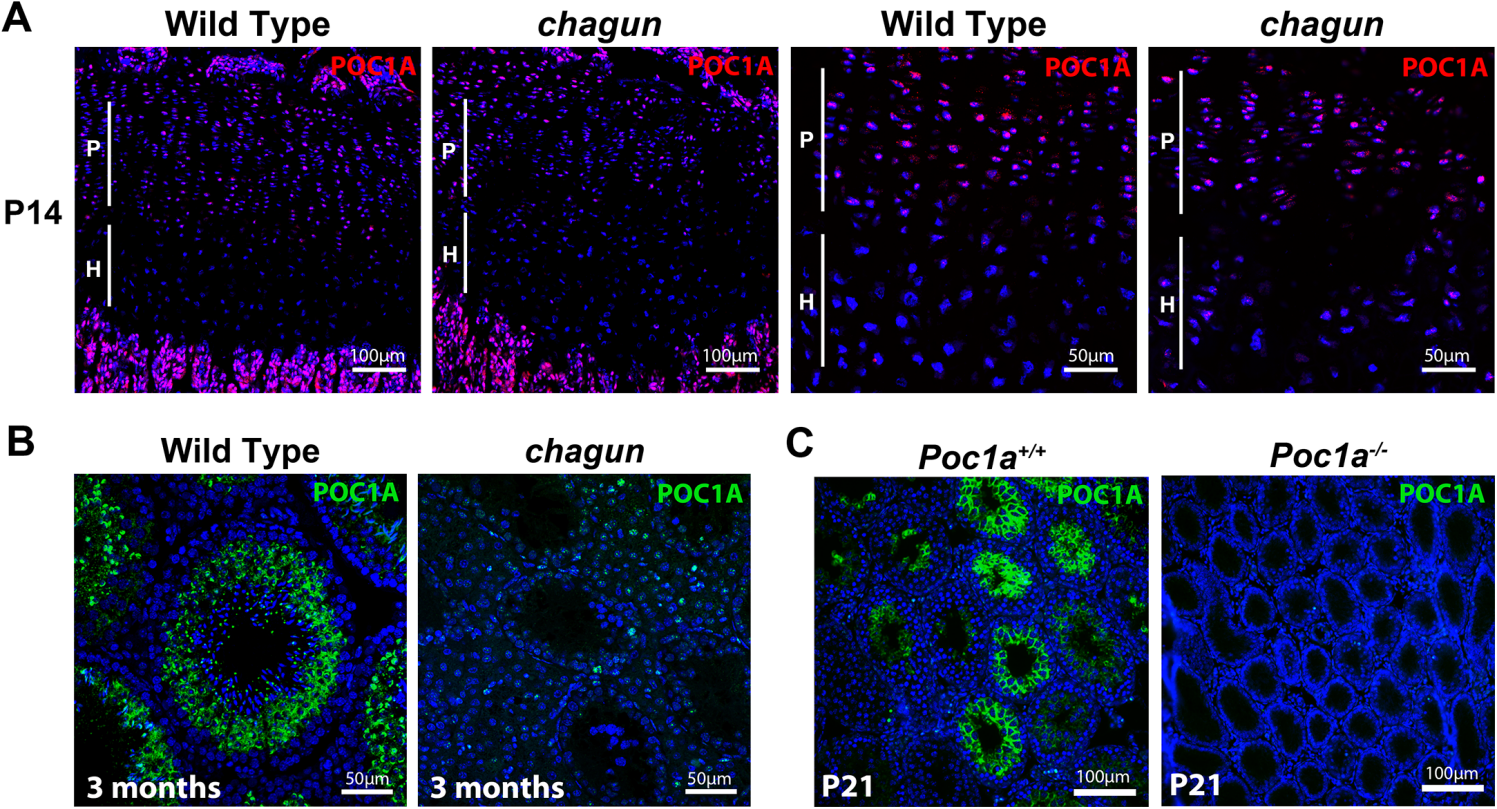
POC1A is expressed in the growth plate and seminiferous tubules. A. POC1A immunostaining of sections from postnatal day 14 (P14) wild type and *Poc1a* ^cha/cha^ mutant tibial growth plates reveals strongest expression of POC1A (magenta) in the proliferative zone. B. POC1A immunostaining was carried out on testis sections from 3 mo old wild type and *Poc1a* ^cha/cha^ mutants. C. POC1A staining was also performed on testis sections from P21 wild type and *Poc1a* knockout mice. Scale bars as indicated.

We performed immunohistochemistry (IHC) using an antibody raised against a 50 amino acid peptide that contains the entire seventh WD40-repeat domain of POC1A. We detected POC1A in the proliferative zone of two-week old wild type (postnatal day 14; P14) tibial growth plates (Fig 4). The discoid chondrocytes in this zone have robust POC1A staining in the cytoplasm. Twenty-one amino acids of the peptide used to raise antibodies are intact in *Poc1a* ^cha/cha^ mutants, and staining is observed in *Poc1* ^cha/cha^ mutant tibia. POC1A is also expressed in seminiferous tubules of the testis, and the immunostaining varies depending on stage of the seminiferous cycle. In wild type mice, POC1A protein is detectable in the cytoplasm of Sertoli cells and as single puncta in spermatozoa and spermatids, which likely represents labeling the centrosome of the spermatids as they begin to form flagella [38–40]. Little POC1A staining is observed in the testes of *Poc1* ^cha/cha^ mutants, suggesting that the mutant protein may be unstable or not retained in this tissue. *Poc1a* knockout mouse testes do not stain with this antibody, confirming the specificity of the antibody for POC1A (Fig 4 and S3 Fig). The expression of POC1A in wild type tibia is consistent with the observed defects in mutant chondrocyte proliferation and expression in wild type male germ cells and Sertoli cells suggests that one or both cell types could be involved in the infertility of male mutants.

### *Poc1a* ^cha/cha^ causes defects in the primary cilium and mitotic spindle in mouse embryonic fibroblasts

The centrosome is the microtubule-organizing center of the cell, and it is responsible for formation of the primary cilium and the mitotic spindle, and organization of the Golgi apparatus [41,42]. The centrosome is comprised of a mother and daughter centriole, and POC1A is a centriolar protein. Therefore, we hypothesized that the *Poc1a* ^cha/cha^ mutation could affect any or all of these centrosome-dependent organelles. To test this prediction, mouse embryonic fibroblasts (MEFs) were isolated independently from three wild type and three mutant embryos and cultured for 24 hours on gelatin-coated coverslips. MEFs were either supplemented with serum to promote cell division and formation of mitotic spindles or serum-starved to facilitate visualization of primary cilia. After the culture period, cells were fixed and immunocytochemistry was conducted.

**Fig 5:**
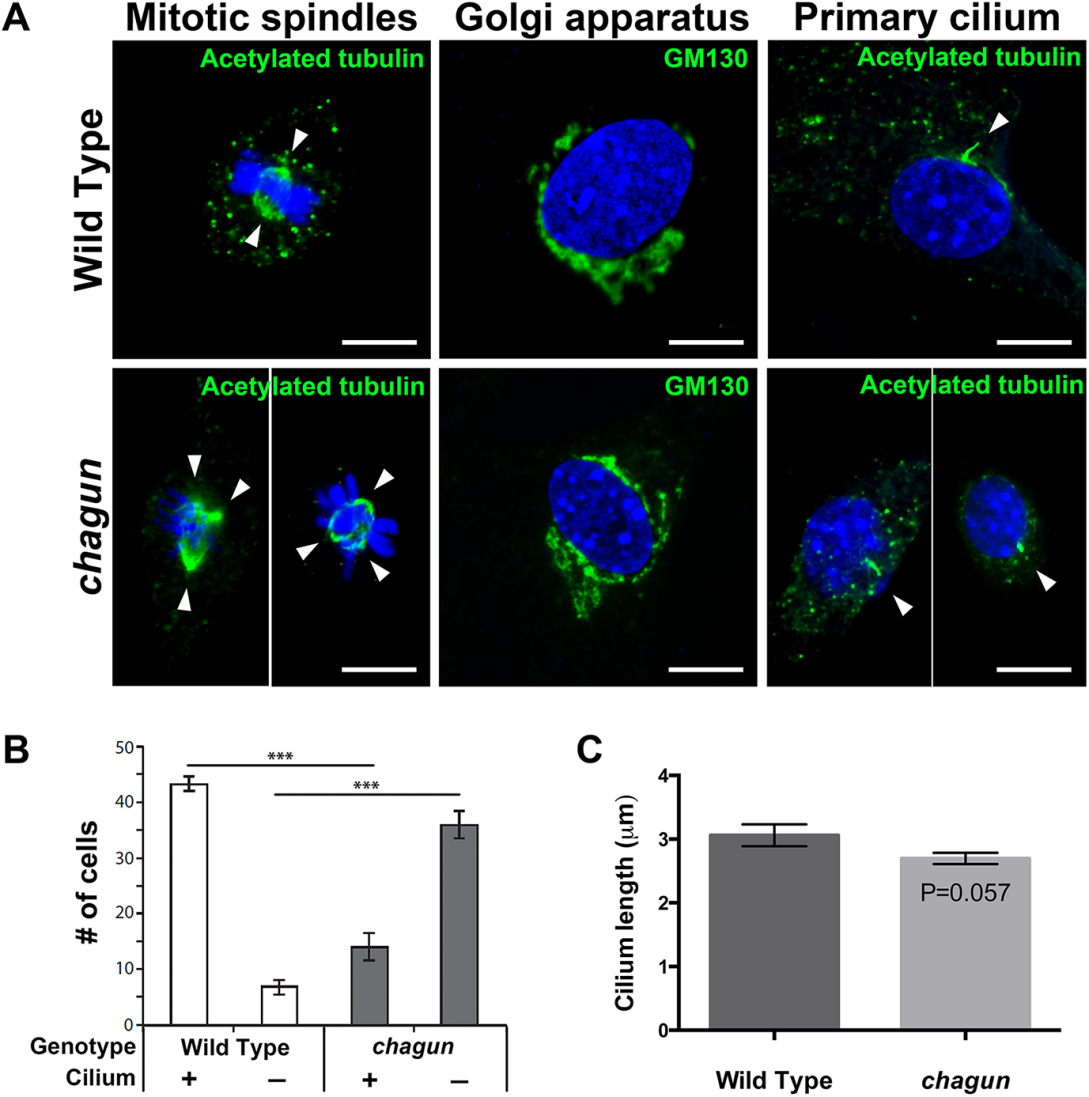
MEFs isolated from *Poc1a* ^cha/cha^ embryos exhibit aberrant mitotic spindles and primary cilia. A. Confocal microscopy images from MEFs of the indicated genotypes incubated with primary antibodies for anti-acetylated tubulin (green) to visualize mitotic spindles (left panels), GM130 to identify Golgi (middle panels) and primary cilia (right panels). All are counterstained with DAPI to visualize nuclei. White arrowheads indicate spindle poles. Scale bar = 10 μm. B. Quantification of the number of anti-acetylated tubulin-stained MEFs of the indicated genotypes with a primary cilium that looks normal in appearance. Using confocal microscopy, 50 cells were counted per slide at 2 slides per sample, and 3 samples per genotype. The mean +/- standard error of the mean are displayed, and data was analyzed by Two-Way ANOVA and Tukey’s Post-Hoc Test were used to analyze statistical significance. The asterisks indicate p < 0.001. C. Quantification of cilium length from a total of 93 *Poc1a* ^cha/cha^ MEFs and 113 wild type MEFs, from 3 lines of each genotype. The mean +/- standard error of the mean are displayed, and the data was analyzed by a generalized estimating equation.

Immunostaining with an antibody against acetylated tubulin, which labels the mitotic spindle and the primary cilium, revealed the effects of the mutation on these two cellular structures. MEFS cultured with serum revealed dividing wild type cells with equal bipolar spindles, as expected (Fig 5). The dividing *Poc1* ^cha/cha^ cells, however, frequently displayed evidence of centrosome amplification, with three or four spindle poles present in a single cell. Centrosome amplification likely results in aneuploidy and cell death. Immunostaining with a GM130 antibody revealed the presence of Golgi near the nuclei of wild type and *Poc1a* ^cha/cha^ mutant MEFs. No obvious differences in Golgi organization were noted. The majority of wild type MEFs cultured without serum had a primary cilium detected by acetylated tubulin immunostaining. Significantly more MEFs from wild type embryos had detectable cilia than *Poc1a* ^cha/cha^ mutants, 87% vs. 28% respectively. The lengths of the measureable cilia were similar in wild types and mutants, 3.06 μm vs. 2.67 μm, respectively. Loss of *Poc1a* function leads to defects in centrosome-mediated processes, including formation of a bipolar spindle and formation or maintenance of the primary cilium in MEFS.

### The *Poc1a* ^cha/cha^ proliferative zone becomes progressively disorganized with age, and displays perturbed chondrocyte polarity and increased cell death

**Fig 6:**
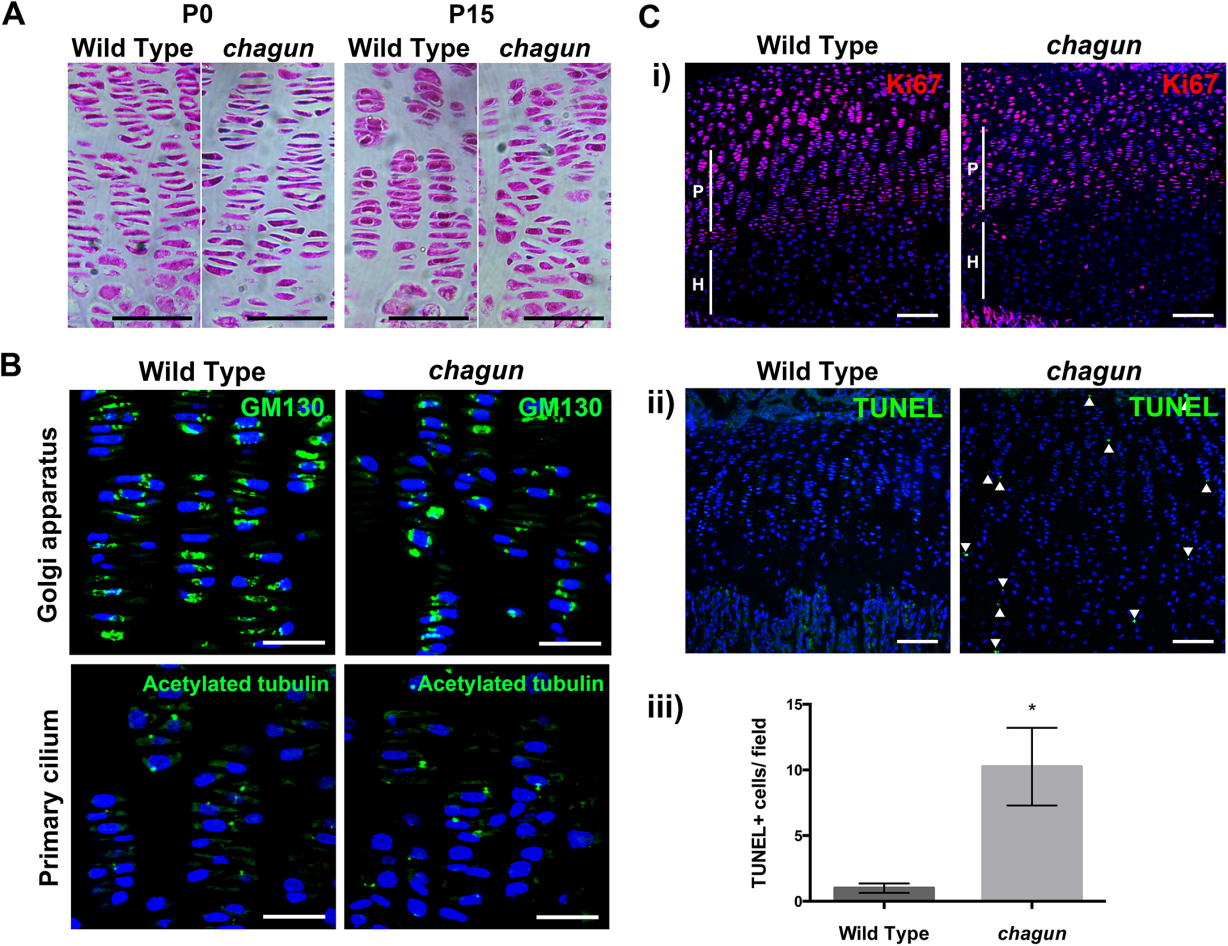
The *Poc1a* ^cha/cha^ long bone growth plates become disorganized. A. Standard hematoxylin and eosin histological staining was carried out on paraffin sections from P0 and P15 tibial growth plates. Scale bar = 50 μm. B. Immunohistochemistry using a primary antibody against the Golgi marker GM130 or acetylated tubulin counterstained with DAPI in wild type and mutant P15 tibia sections. Scale bar = 25 μm. C. Immunohistochemistry for primary antibodies against the proliferation marker Ki67 (i) or cell death marker TUNEL (ii), with quantification of the number of TUNEL-positive cells per field of view in wild type and *chagun* tibia (iii). Scale bar = 100 μm.

The growth plates of vertebrae and long bones of *Poc1a* ^cha/cha^ mice are obviously disorganized [26]. Histology of tibiae from the neonatal period to P21 indicates that the growth plate becomes progressively more disorganized with age. At P0, the mutant growth plates look normal (Fig 6), but by P15, the coin stack structure of the chondrocytes in the proliferative zone begins to deteriorate in mutants, and some mutant chondrocytes have a rounded, rather than flat, appearance. By P21, *Poc1a* ^cha/cha^ tibial growth plates are even more severely disorganized (S4 Fig)

The subcellular position of the Golgi within the columnar chondrocytes of the proliferative zone can provide information about the polarity of the cell [3,43]. The Golgi is typically located to the right or left of the nucleus in chondrocytes, towards the edge of the coin stack column. To test whether the Golgi are positioned normally in *Poc1a* ^cha/cha^ chondrocytes, immunohistochemistry was conducted on sections of the tibial growth plate utilizing primary antibodies against the Golgi marker GM130. The lateral subcellular positioning of the Golgi is evident in both wild type and mutant cells in the proliferative zones (Fig 6). The mutants have more nuclei that are more rounded, and the mutant cells appear mal-rotated relative to the cellular column. This indicates that the chondrocytes in *Poc1a* ^cha/cha^ mice exhibit some polarized Golgi localization, but fail to maintain cell shape or ability to re-align after cell division.

The primary cilia of chondrocytes in the columns of the proliferative zone are oriented in the direction of the column—pointing toward the resting or hypertrophic zone [3,44,45]. We conducted immunohistochemistry of tibia using a primary antibody against acetylated tubulin on sections from P15 wild type and *Poc1a* ^cha/cha^ mutant mice to test whether primary cilia were properly oriented in the mutant growth plate. Wild type growth plates displayed normal orientation of the primary cilia within their columns. Primary cilia were detected in the *Poc1a* ^cha/cha^ growth plate, and some of them are oriented properly (Fig 6).

Formation of multipolar spindles could lead to premature chondrocyte cell death. Ki67 staining is similar in *Poc1a* ^cha/cha^ mice, being largely expressed to the proliferative zone. TUNEL staining of P15 tibial sections demonstrated a substantial increase (10.3 fold, p = 0.002) in TUNEL-positive nuclei in the *Poc1a* ^cha/cha^ growth plate (Fig 6). Together, these data indicate that the proliferating chondrocytes in *Poc1a* ^cha/cha^ mice fail to maintain their cell shape, fail to intercalate properly after cell division, and undergo a significantly higher rate of cell death.

### Germ cell maturation arrest during postnatal life in *Poc1a* ^cha/cha^ males leads to infertility

**Fig 7:**
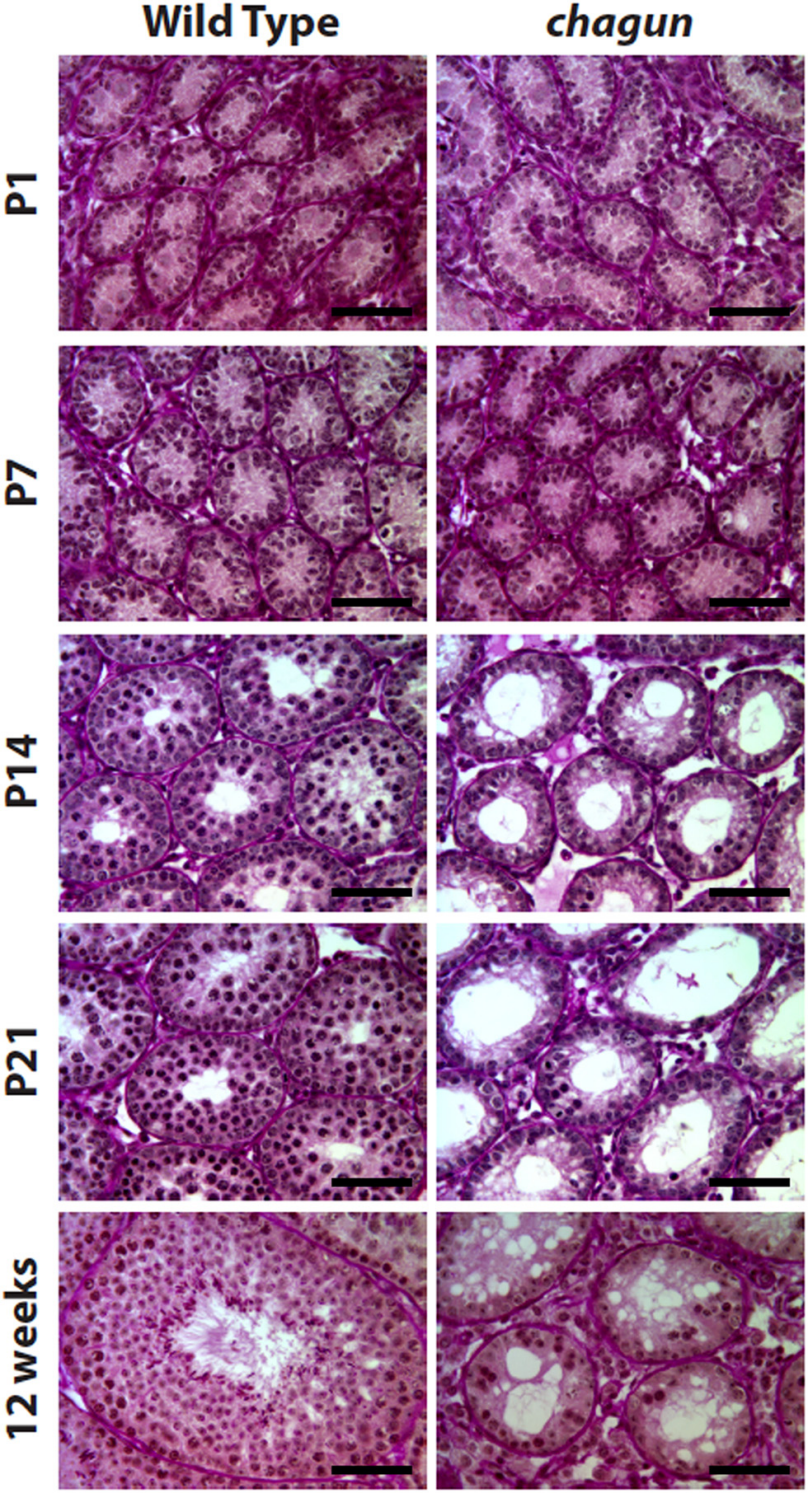
*Poc1a* ^cha/cha^ males exhibit progressive germ cell loss. Paraffin sections of testes tissues collected from wild type and *Poc1a* ^cha/cha^ mice at the indicated ages were stained with Periodic acid-Schiff reagent. At P1 and P7 *Poc1a* ^cha/cha^ tubules resemble wild type seminiferous tubules, but from P14 through 12 wks, the *Poc1a* ^cha/cha^ tubules are obviously abnormal. (N = 3 animals of each genotype at each age, scale bar = 50 μm).

*Poc1a* ^cha/cha^ males are infertile. In adult mutant males, the latest stage of spermatogenesis that is detected is the pachytene stage, and hypogonadism ensues with age [26]. However, the time of initial germ cell disruption during postnatal development was not previously determined. To address this, we conducted histological analysis of the testis in normal and *Poc1a* ^cha/cha^ mutants from birth to adulthood. Mutants were identified by *Poc1a* exon 8 genotyping and periodic acid-Schiff (PAS) histological staining was conducted on cross-sections of testes from males at postnatal day 1 (P1), P7, P14, P21, and 12 weeks (Fig 7). Immediately after birth (P1), the number of germ cell and Sertoli cell nuclei per tubule and the diameter of seminiferous cords in testis cross-sections from *Poc1a* ^cha/cha^ males were indistinguishable from wild type counterparts, indicating that during embryonic development the proliferation and migration of germ cells to the genital ridge to form testis cords occurs normally. At P7, seminiferous cords from *Poc1a* ^cha/cha^ males are still morphologically similar to wild type; however, the number of γH2AX positive cells is reduced in mutant males, indicating loss of spermatocytes at the earliest stages of meiosis (S5 Fig). By P14 the morphology of seminiferous tubule cross-sections from *Poc1a* ^cha/cha^ mutants are dramatically different from controls. Progression of germ cell maturation to the pachytene spermatocyte stage is clearly evident in tubules of wild-type mice at P14. In contrast, only a few spermatocytes are detected in the mutant tubules. At P21, germ cell maturation is still arrested in testes of *Poc1a* ^cha/cha^ mutants, and most of the germline appears to be lost in some of the tubules. The testis of sexually mature 12 week old wild type mice contain all stages of spermatogenesis, including undifferentiated and differentiating spermatogonia, primary and secondary spermatocytes, and round and elongating spermatids. In contrast, very few germ cells are present in testis cross-sections from 12 wk *Poc1a* ^cha/cha^ mutants. Although some tubules contain a few leptotene or pachytene spermatocytes, most tubules appear to be devoid of all differentiating germ cells and contain spermatogonia only. None of the tubules in *Poc1a* ^cha/cha^ adult mice contain secondary spermatocytes or post-meiotic round/elongating spermatids. Taken together, these observations demonstrate that the *Poc1a* ^cha/cha^ testis phenotype is caused by a postnatal defect in spermatogenesis beginning at the pre-leptotene stage of meiosis, rather than impairment of testis cord formation during embryogenesis.

### Spermatogonial stem cell transplant reveals defects in mutant Sertoli cells and germ cells

**Fig 8:**
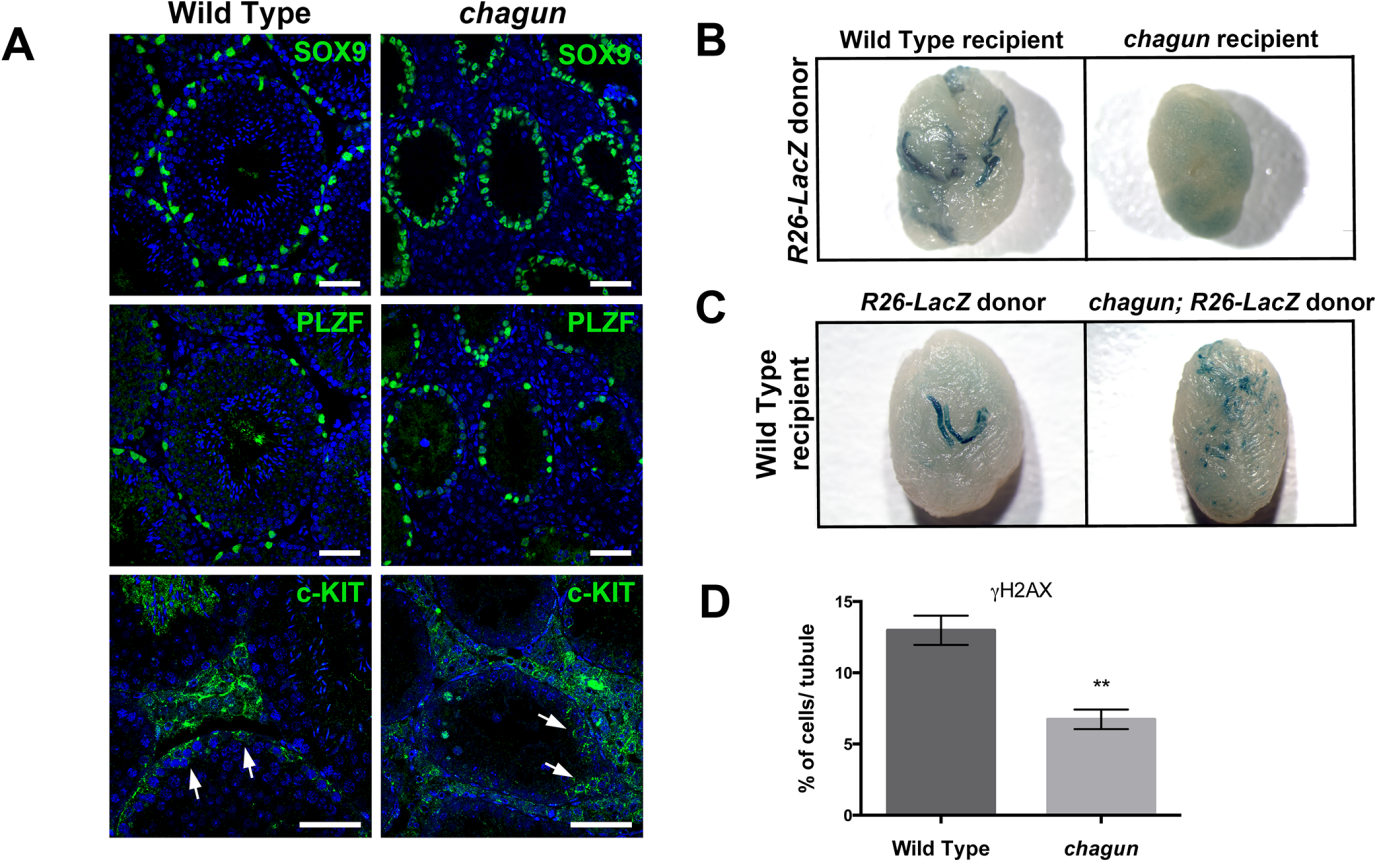
Spermatogonial stem cell (SSCs) transplants reveal Sertoli cell and germ cell defects in *Poc1a* ^cha/cha^ mice. A. Testis sections from 3 mo wild type and *Poc1a* ^cha/cha^ mice were immunostained for SOX9, a Sertoli cell marker, and PLZF, an undifferentiated spermatogonial marker, and counterstained with DAPI to visualize nuclei (N = 3 animals of each genotype, scale bar = 50 μm). B. Testes from busulfan treated wild-type recipient or *Poc1a* ^cha/cha^ mutant recipients ~3 months after transplantation with wild-type LacZ labeled SSCs. The testes (N = 8) of recipient mice (N = 4) were transplanted for each genotype. C. Testes from busulfan-treated wild-type nude recipient mice ~3 months after transplantation with wild type LacZ-labeled SSCs or *Poc1a* ^cha/cha^ SSCs labeled with LacZ. For both B and C, blue stained areas in recipient testes represent colonies of donor-derived spermatogenesis generated from engrafted SSCs. D. Quantification of γH2AX-positive cells in seminiferous tubules of 3 wild type and 3 *Poc1a* ^cha/cha^ testes at P7.

Sertoli cells function to nourish the germ cells, phagocytose cytoplasmic remnants during spermatogenesis, and promote the translocation of the developing germ cells from the base of the tubule to the lumen. Impaired spermatogenesis could result from defects in Sertoli cells, germ cells, or both. To assess the relative contributions of Sertoli cells and germ cells, we used immunohistochemistry to detect these cell types, with primary antibodies against SOX9, PLZF (promyelocytic leukemia zinc finger protein) and c-KIT. SOX9 is a marker of Sertoli cells. PLZF marks the undifferentiated spermatogonial population that consists of spermatogonial stem cells (SSCs) and progenitor spermatogonia that are primed to undergo differentiation [46,47]. The expression of c-KIT indicates that spermatogonia have initiated differentiation [48]. At P14 the number of Sertoli cell nuclei per tubule cross-section is not different in wild type and *Poc1a* ^cha/cha^ males (WT = 23.3±2.3 cells/tubule vs *Poc1a* ^cha/cha^ = 26.6±4.0 cells/tubule, p = 0.52). At 3 months of age, the number of Sertoli cells per tubule is also not diminished in *Poc1a* ^cha/cha^ testis. The apparent increase in Sertoli cell number per tubule in mutants (Fig 8A) is likely due to reduced tubule size and defective spermatogenesis in mutants that arbitrarily causes a greater concentration of Sertoli cells per mm of tubule length. The number of PLZF+ cells per testis section does not differ in wild type and mutant testes at 3 mo (Fig 8A), or at earlier ages (S6 Fig). The quantitation of undifferentiated spermatogonia (i.e. PLZF+ cells) in wild type vs *Poc1a* ^cha/cha^ testis at each age was as follows: P7: 8.1 ±0.4 vs 4.8±1.2, p = 0.07. P14: 13.4±1.6 vs. 9.5±0.2, p = 0.07. P30: 5.9±0.9 vs 9.5±1.4, p = 0.1. The presence of c-KIT+ spermatogonia indicates that spermatogonial differentiation occurs in both wild type and *Poc1a* ^cha/cha^ testes (Fig 8).

The histone marker γH2AX is rapidly phosphorylated in response to double strand breaks, and it recruits DNA damage response factors. It is prominently expressed in all intermediate and B spermatogonia and in pre-leptotene to zygotene spermatocytes. While the proportion of tubules in *Poc1a* ^cha/cha^ testes with γH2AX staining is similar to wild types at P7 (61%±14% vs 65%±8%, not significant), there is a major reduction (1.9 fold) in the number of γH2AX-positive cells within these tubules in the *Poc1a* ^cha/cha^ mutant testes: 12.98% vs 6.73%, respectively (Fig 8, Panel D). The defect is much more severe at P14 (S5 Fig). This suggests a loss of germ cells early in meiosis at the pre-leptotene and zygotene stages, resulting in some spermatogonia at the pachytene stage and none at later stages. Condensation of chromosomes and formation of synaptonemal complexes takes place at these early meiotic stages.

Next, we utilized spermatogonial stem cell (SSC) transplantation to determine whether the spermatogenesis defect was attributable to poor Sertoli cell function, intrinsic defects of the germ cells, or both [49]. In the first set of experiments, we transplanted Rosa-LacZ marked wild type spermatogonia into the seminiferous tubules of wild type recipients pre-treated with busulfan to eliminate the endogenous germline, or alternatively into adult *Poc1a* ^cha/cha^ mutant males with impaired spermatogenesis (N = 4 recipients and 8 testes per genotype). Approximately 3 months after transplantation, X-gal staining revealed that the wild type donor SSCs generated colonies of donor-derived spermatogenesis in wild type recipient tubules, as expected. No colonization was detected in any of the *Poc1a* mutant testes (Fig 8B). The failure of *Poc1a* mutant testes to support the engraftment of wild-type SSCs is not attributable to the presence of residual mutant SSCs because donor SSCs can colonize normal testes with normal spermatogenesis occurring, albeit at a much lower rate [50]. These results are consistent with a Sertoli cell defect in the *Poc1a* mutants that could impair the homing ability of transplanted wild-type SSCs and/or re-establishment of colonies of continual spermatogenesis. Impaired homing seems unlikely to be the major cause because a primary spermatogonial population is established during neonatal development in *Poc1a* ^cha/cha^ mutant testes. Thus, migration of germ cells from the lumen of the cords to the basement membrane during postnatal development occurs normally in *Poc1a* ^cha/cha^ males.

For the second set of experiments, we crossed the Rosa-LacZ marker into the *Poc1a* ^cha/+^ stock to produce *Poc1a* ^cha/cha^ males with LacZ marked germ cells. The marked, mutant spermatogonia were transferred into the seminiferous tubules of busulfan-treated wild type recipient nude mice. As a control, Rosa-LacZ marked wild-type spermatogonia were transferred to identical recipients at the same time. As expected, the wild-type SSCs generated robust colonies of spermatogenesis with uniform blue staining in testes of all recipients (N = 4 recipient mice and 8 testes). In contrast, only small patches of spermatogenesis with non-uniform blue staining were generated from *Poc1a* ^cha/cha^ mutant SSCs, consistent with blunted expansion of colonizing SSCs and arrested spermatogenesis. Transplantation of mutant cells did not generate densely stained colonies of donor-derived spermatogenesis in any recipient transplanted testes (N = 4 recipients and 8 testes). Taken together, these findings suggest that the SSCs of *Poc1a* ^cha/cha^ mutants are capable of initiating a colonization of a wild type microenvironment, but that intrinsic defects in the germ cells cause arrested spermatogenesis, even with wild type Sertoli cell support. These results are consistent with the idea that POC1A expression in both germ cells and Sertoli cells is important for normal testicular function.

## Discussion

### The *chagun* mutation is a LINE-1 retrotransposon-mediated insertion of a processed *Cenpw* transcript in exon 8 of *Poc1a*

We report the discovery of the genetic basis of skeletal dysplasia and spermatogenic failure in *chagun* mutants. Insertion of a processed *Cenpw* transcript into exon 8 of the gene that encodes protein of the centriole 1A (*Poc1a*) causes elimination of a portion of one of the seven highly conserved WD40-repeat domains and abrogates function. We conclude this based on: 1) identification of an exonic insertion that causes skipping of exon 8 in *Poc1a* mRNA transcripts, 2) co-segregation of this *Poc1a* mutation with the *chagun* mutant phenotype, 3) the lack of unique insertions, deletions, or coding region mutations in other genes within the *chagun* critical interval, 4) successful BAC transgenic rescue of the mutant phenotype, 5) recapitulation of the mutant phenotype with a null, lacZ knock-in allele, and 6) expression of *Poc1a* in the affected tissues. Taken together, this provides compelling evidence that the insertion in *Poc1a* causes the *chagun* phenotype.

Failure to detect the insertion by exome sequencing is probably attributable to the fact that all of the exon 8 genomic DNA sequence is still present in the mutants. The insertion of the *Cenpw* processed transcript increases the size of exon 8 from 69 bp to 564 bp, and increased exon size can cause skipping [51]. Skipping of exon 8 maintains the *Poc1a* open reading frame, and the predicted POC1A mutant protein lacks 23 amino acids in the most C-terminal, highly conserved WD40 repeat domain. POC1A has seven WD40 repeats that are expected to form a seven bladed, circular beta propeller structure. The lesion in the 7^th^ repeat likely causes a failure of the blades to interlock. The mutant POC1A protein is readily detectable in tibia but not testis of *Poc1a* ^cha/cha^ mice, suggesting tissue specific effects on protein stability.

The *Cenpw* cDNA insertion appears to be a LINE-1 mediated event because it is flanked by a 15 bp target site duplication. In addition, the sequence 5’ TTTC|A in wild type exon 8 matches the ORF2 consensus (reviewed in [29]). Cleavage between the C and A at these sites permits target-primed reverse transcription by ORF2 reverse transcriptase [30,52,53], resulting in insertion of the transcript into exon 8 of *Poc1a* by the LINE-1 encoded proteins [54,55]. We are aware of only two other examples of LINE-1-mediated mutagenesis that involve non-LINE1 transcripts [56–58]. An insertion of a retrogene into an intron of FGF4 causes short stature in 19 dog breeds, and early dog breeders apparently selected for it. The insertion causes atypical expression of FGF4 in chondrocytes, rather than loss of function that we observed in *Poc1a* ^cha/cha^ mice. Chronic granulomatous disease, an immunodeficiency disorder in humans, is caused by a LINE-1 mediated insertion of a partially processed transcript into an intron of the *CYBB* gene, which encodes a cytochrome that is essential for phagocytosis by leukocytes. The insertion causes loss of function; it disrupts splicing, resulting in incorporation of a novel exon and premature termination. Processed pseudogenes have also been detected in cancer genomes [59]. The difficulty in identifying the *chagun* mutation by exome sequencing suggests that the paucity of examples of LINE-1 mediated mutagenesis could, in part, due to alignment issues with the programs used to analyze exome sequencing data, resulting in ascertainment bias.

### The *Poc1a* ^cha/cha^ mutant models a human skeletal dysplasia syndrome

Two mutations in *POC1A* (p.Arg81X and p.Leu171Pro) were recently reported in separate consanguineous pedigrees with severe short stature and craniofacial abnormalities [21,22]. Sarig, Sprecher, and colleagues labeled the syndrome a primordial dwarfism called SOFT Syndrome (short stature, onychodysplasia, facial dysmorphism, and hypotrichosis) [21]. Shaheen, Alkuraya and colleagues reported the p.Leu171Pro mutation, and they noted global developmental delay including cognitive impairment in some affected individuals, but they did not observe hair or nail defects [22]. We observed no fur or nail abnormalities in the *chagun* mutants or ES derived *Poc1a* null mutants. There was no discussion of hypogonadism or fertility of the human subjects in either report. The *POC1A* mutation p.Arg81X permits significant read through translation, which left open the possibility that it is a hypomorphic allele. The growth insufficiency, however, is severe in patients with either mutation. Mice homozygous for the ES-derived null allele of *Poc1a* have the same growth defect, skeletal dysplasia, and testicular features as *Poc1a* ^cha/cha^ mice. This indicates that *Poc1a* ^cha^ is a loss of function allele. The variable secondary features in the two families could be due to functional differences between the two alleles, contributions from mutations in other genes, or genetic modifiers. Both mutant mice exhibit severe growth abnormalities and craniofacial dysmorphism, and provide excellent models for the major features of the human syndrome.

### Loss of *Poc1a* function leads to defects in spindle poles and primary cilia in embryonic fibroblasts

In metazoans, the centrosome regulates organization of microtubules and progression of the cell cycle [60]. Two centrioles, cylindrical, tubulin-rich structures that, together with additional peri-centriolar material, form a single centrosome in each cell. The centrosome is typically located centrally near the nucleus of the cell, but it moves to the leading edge of polarized, migrating cells. At the G1 to S transition, the centrosome begins to duplicate, and after the G2 to M transition, the mother and daughter centrosomes migrate to opposite poles of the cell and form the mitotic spindles. The centrosomes are not strictly required for spindle formation, but they are believed to enhance its efficacy and ensure the fidelity of cell division. While cells are in the quiescent state the centrosome migrates to the cell surface and forms the basal body of the primary cilium. The centrosome and Golgi apparatus are juxtaposed during interphase and are thought to interact functionally for directional protein transport.

Proteome analysis in *Chlamydomonas* and *Tetrahymena* identified eighteen different proteins of the centriole, including *Poc1*, which is a core component of the centriole and basal body in all organisms with motile cilia [61]. Vertebrates have two genes, *Poc1a* and *Poc1b*, which are broadly expressed, exhibit 49% amino acid identity, and have overlapping function in cell culture [62]. Embryonic fibroblasts from *Poc1a* ^cha/cha^ embryos exhibit a number of anomalies in centrosome-mediated processes. The majority of dividing mutant fibroblasts form aberrant mitotic spindles, and the primary cilia are infrequent and abnormally shaped. We did not notice any obvious defects in the organization of the Golgi. We observed binuclear *Poc1a* ^cha/cha^ MEF cells, which are likely indicative of failed cytokinesis, similar to observations of *Poc1* dysfunction in Tetrahymena [63]. Skin fibroblasts from human patients with POC1A dysfunction also had abnormalities in mitotic spindle polarity and the formation and length of the primary cilium [21], and although centrosome ultrastructure was normal, centrosome number increased and Golgi trafficking was impaired [22]. Knock down of *Poc1a* in cells leads to an inability to recruit markers of a mature centrosome [62]. Taken together, these data suggest that although POC1A deficient fibroblasts can form normal looking centriolar structures, the recruitment of proteins to the centriole is defective, causing abnormal centrosome function and increased number of centrosomes per cell. This leads to multipolar spindles and failed cytokinesis. The centrosome abnormalities also lead to defects in cilia formation and/or maintenance. *Poc1a* ^cha/cha^ chondrocytes likely have defects in centrosomes, spindle poles and cytokinesis similar to those observed in *Poc1a* ^cha/cha^ embryonic fibroblasts and *POC1A* patient fibroblasts [21,22].

### *Poc1a* is required for the morphology, alignment and survival of proliferating chondrocytes

Cell division in the growth plate is an intriguing process in which chondrocytes undergo polarized cell division orthogonal to the main direction of bone growth and spread back over one another to form columnar structures comprised of disc-shaped chondrocytes [6,64]. The growth plates of newborn *Poc1a* ^cha/cha^ mutants have normal organization into zones and normal morphology of proliferating chondrocytes, but by puberty the proliferating chondrocytes are losing the typical coin-stack structure, and their shape is abnormally rounded rather than flat. As the proliferating cells become more disorganized, apoptosis is increased, and the final size of the proliferative zone is reduced. Although mutant chondrocytes can produce cilia and exhibit polarization of the Golgi, the function of the centrosomes and cilia are likely impaired. This view is supported by the similar chondrocyte disorganization and abnormally round cells in mice with disruption of *Kif3a*, a kinesin II motor complex protein required for intraflagellar transport and cilia formation [3]. Cilia defects are known to affect the Wnt/Planar cell polarity pathway, which is important for cellular shape, migration and organization [42,65,66]. The primary cilium is also important in the cellular response to mechanical cues that govern growth plate structure. For example, disruptions of the osteoblast primary cilium affect mechanoresponsiveness, mesenchymal cell differentiation [67], and the response to mechanical loading [68,69]. Thus, the centrosomal defects in *Poc1a* mutants could account for abnormal microtubule organization affecting chondrocyte morphology, aneuploidy leading to increased cell death, and poor cilia function influencing cell alignment at the growth plate.

### *Poc1a* is required for both Sertoli cell function and germ cell differentiation

Testosterone levels, seminal vesicle development, and Leydig cell structure are all normal in *Poc1a* ^cha/cha^ males [26], suggesting normal function of the hypothalamic-pituitary-gonadal axis. The germline is established normally in *Poc1a* ^cha/cha^ males during embryogenesis and early neonatal life, as indicated by normal testicular morphology and PLZF-positive spermatogonia. Hypogonadism and infertility arise later in postnatal development when spermatogonia normally undergo a transition from mitotic to meiotic divisions. Defects in germ cells and Sertoli cells contribute to the infertility. Mutant SSCs transplanted into wild type hosts are capable of initial colonization, but they do not establish colonies typical of normal spermatogenesis. There are examples of spermatocytes with chromosome mispairing or DNA repair defects that are arrested at the pachytene checkpoint and undergo apoptosis [70]. In the current study, we extended the assessment of spermatogenic defects and discovered a reduction of meiotic cells at P7 in *Poc1a* ^cha/cha^ males compared to wild type counterparts, which is when pre-leptotene spermatocytes first arise in the male germ cell lineage. Also, we found that the number of spermatocytes is greatly reduced in *Poc1a* ^cha/cha^ males at P14 when pachytene stage cells first arise. Together, these findings indicate that defects in germ cell maturation begin to arise at the earliest stages of meiosis but become more pronounced as meiotic progression ensues, eventually leading to elimination of a majority of the population. We predict that *Poc1a* mutant spermatocytes are aneuploid, which triggers apoptosis at the pachytene checkpoint. Also, our findings suggest that *Poc1a* ^cha/cha^ Sertoli cells do not support the engraftment of wild type SSCs or development into mature sperm, possibly due to defects in the microtubule organizing center and/or failure to secrete factor(s) essential for spermatogonial survival and/or germ cell maturation.

The arrangement of microtubules in Sertoli cells is unique and highly specific to this cell type [71]. Defective centrosomes lacking POC1A could disrupt microtubule organization, leading to defective SSC niches [72] and/or an inability to support cells at later stages of spermatogenesis. The inability of wild-type SSCs to colonize *Poc1a* ^cha/cha^ testes suggests that the mutation disrupts the ability of the testis microenvironment to support engraftment of normal SSCs in the niche at the basement membrane. Compromised trafficking of components required for the germ cells, or even the ability of the Sertoli cells to support the development of spermatocytes and spermatids could contribute to impaired spermatogenesis in *Poc1a* ^cha/cha^ mutant testes. Immature or multiple centrosomes could impair the ability of Sertoli cells to support differentiation and development of spermatozoa from SSCs.

### POC1A and POC1B have unique and overlapping functions

*Poc1a* and *Poc1b* are both expressed in testis and bone (Fig 4 and S7 Fig), and have functional overlap in cell culture [62,63]. Mutations in human *POC1B*, however, cause a very different disorder. Homozygotes for the p.Arg106Pro mutation in *POC1B* have severe, syndromic retinal ciliopathy with defects in kidney and cerebellar function [73]. Individuals with p.Gln67del mutations also have recessive, non-syndromic cone rod dystrophy [74]. The basis for these tissue-specific effects is not clear.

### Summary

A loss of function of *Poc1a* causes skeletal dysplasia and male infertility in *chagun* mice. Insertion of a processed cDNA causes exon skipping and predicted deletion of 23 highly conserved amino acids necessary for the structural integrity of the protein. POC1A is expressed in the growth plate of long bones and the seminiferous tubules of the testis, the tissues with the most obvious cellular phenotypes. Several processes contribute to the growth defect. First, proliferating chondrocytes undergo enhanced cell death, likely due to multipolar spindle formation. Second, rapidly proliferating chondrocytes fail to maintain the characteristic flattened cell shape and fail to intercalate into a cellular column after cell division, consistent with defective cilia function. Male infertility is a consequence of the inability of Sertoli cells to support germ cell development, as well as germ cell defects in spermatogenesis. The *Poc1a* ^cha/cha^ mouse has provided information on the molecular mechanism underlying clinical features of human patients, and highlights male fertility as a potential area of interest for clinicians examining adult male patients with mutations in *Poc1a*. The *Poc1a* ^cha/cha^ and *Poc1a* ^tm1(KOMP)Mpb^ mice are valuable tools for studying the molecular mechanisms of dwarfisms and for testing therapeutic interventions.

## Materials and Methods

### Mice

The *chagun* mutation arose spontaneously on the DBA/2J strain in Dr. Linda D. Siracusa’s laboratory at Thomas Jefferson University (Philadelphia, Pennsylvania). The mice were transferred to the University of Michigan (Ann Arbor, Michigan). Recently they have been maintained on a hybrid background consisting of C57BL/6J and DBA/2J background strains, and also outcrossed to the FVB/NJ strain for routine maintenance.

The BAC RP24-384G5 was purchased from Children’s Hospital of Oakland Research Institute (CHORI). Transgenic mice carrying this BAC were generated by the University of Michigan Transgenic Animal Model Core [36].

Embryonic stem cells containing the *Poc1a* ^tm1(KOMP)Mbp^ mutation, CSD45930, were purchased from the KOMP (University of California, Davis, CA). These stem cells, JM8A3.N1, were derived from the C57BL/6N-A^tm1Brd^ strain. Two targeted clones were used: DEPD00572_5_B12 (Poc1a_B12), and DEPD00572_5_C09 (Poc1a_C9). These stem cells were injected into blastocysts and transferred to pseudopregnant surrogate mothers by the University of Michigan Transgenic Animal Model Core. Chimeras were mated to C57BL/6J females to obtain germline transmission.

All mice were housed in a specific pathogen free facility with 12-h light, 12-h dark cycle in ventilated cages with unlimited access to tap water and Purina 5020 chow. All procedures using mice were approved by the University of Michigan Committee on Use and Care of Animals (UCUCA), and the Washington State University Institutional Animal Care and Use Committee (IACUC), and all experiments were conducted in accordance with the principles and procedures outlined in the National Institutes of Health Guidelines of the Care and Use of Experimental Animals. Euthanasia was conducted by CO_2_ inhalation, except for newborn mice that are unresponsive to this method. Decapitation was used for those animals. Surgery was conducted with approved anesthetics. Experienced veterinary care was provided.

### Genotyping protocols

Two protocols were used to genotype for *chagun*. Before the *chagun* mutation was uncovered, the genotype at *cha* was inferred based on polymorphic flanking markers. Primers were designed to amplify regions of genomic DNA flanking the *chagun* critical interval that contained informative SNPs. These SNPs differ between the mutant (DBA/2J) and the wild-type (FVB/NJ) backgrounds: rs30174769, rs29637716. These amplification products were digested with Bsr1 endonuclease and run on a 2% agarose/tris-boric acid-EDTA gel to visualize the different bands that segregated differentially between the two background strains. After the mutation was uncovered, mutants, heterozygous, and wild-type animals were distinguished using primers designed to detect the presence of the insertion from flanking sequence (two potential products; a small wild-type allele, and the larger *chagun* allele with the insertion).

Animals from the BAC transgenic rescue experiment were genotyped using the polymorphic SNP markers, and at least two additional sets of primers that amplify products specific to the BAC backbone. All primers anneal optimally at 60°C. The forward (Fwd) and reverse (Rev) primer sequences are:

rs30174769 Fwd: 5’-TGAGCGCCAACACAGTAAAAA-3’
rs30174769 Rev: 5’-GTGGGAGCAAGAGACAAAGAGAT-3’
rs29637716 Fwd: 5’-GCGGGGTGCCTGGGTCTCC-3’
rs29637716 Rev: 5’-TCATACAGCACACGCACTTACACA-3’
T7 Fwd: CGGGGCACATTTCATTACCTCTTT
T7 Rev: GCTCGGCATGCACAACTGATTA
SACBII Fwd: TTGACGGAGACGGAAAAACATA
SACBII Rev: ACCGCGTGAATCAGTGAACAAGTA
WT and *chagun* allele Fwd: 5’-TGTCCCACTGCCACTGCCACTCA-3’
WT and *chagun* allele Rev: 5’-GGAAGACTCGCCCCACAGGACTCA-3’

Animals were genotyped for the presence of the *Poc1a* ^tm1(KOMP)Mbp^ mutation by PCR with primers for LacZ and primers in *Poc1a* exons 6 and 7, which are not present in the *Poc1a* ^tm1(KOMP)Mbp^ mutant allele. Primers for LacZ amplification: Fwd: ATCCTCTGCATGGTCAGGTC Rev: CGTGGCCTGATTCATTCC. Amplification was at 94C for 3 min followed by 35 cycles of 94C 30sec, 58C 30 sec, 72C 30sec, followed by 72C 10min. Primers for detection of wt *Poc1a*: Fwd: TCTGCTTTGCGGTGTACGAA Rev: TTGGGTAGGGTGGGGTACAT. The conditions for this PCR are 92C for 2 min followed by 30 cycles of 92C 10sec, 57C 30sec, 72C 30sec.

### Targeted genomic DNA capture

Two different capture approaches were taken. The Broad Institute conducted genome-wide exome capture and sequencing (Mutant Mouse Resequencing Project, The Broad Institute of MIT and Harvard, Cambridge, MA). Regional targeted enrichment was performed using custom capture probes targeting non-repetitive sequences within the *chagun* critical interval, and targeted enrichment libraries were generated (Roche/Nimblegen, Madison, Wisconsin). Genomic DNA samples from both a heterozygote (known carrier) and a *chagun* mutant were used to generate these targeted enrichment libraries, which were indexed separately to allow for multiplexed high-throughput sequencing (IlluminaHiSeq) by the University of Michigan DNA Sequencing Core. DNA Sequence reads were aligned to the B6 reference genome (mm9) using BWA [6]. Custom perl scripts were used to identify the location of potential insertional elements, using the paired-end read mapping data, and these were manually reviewed using Broad Institute’s Integrative Genomics Viewer to visualize and evaluate the DNA sequence data [28].

### PCR amplification of genomic DNA

The region around *Poc1a* exon 8 was amplified by PCR of genomic DNA using the following primer sequences: Fwd: 5’-TGTCCCACTGCCACTGCCACTCA-3’, and Rev: 5’-GGAAGACTCGCCCCACAGGACTCA-3’. Optimal annealing temperature: 60C. PCR was conducted with the GoTaq DNA Polymerase and buffers provided by the distributor (Promega). Sanger sequencing was completed by the University of Michigan DNA Sequencing Core.

### Extraction of RNA and generation of cDNA via reverse transcription

Extraction of total RNA was completed with the RNAqueous 4-PCR Kit (Ambion) according to the manufacturer’s instructions. The total RNA was utilized to generate cDNA with Oligo dT Primers (Invitrogen/Life Technologies) and Superscript II Reverse Transcriptase (Invitrogen/Life Technologies) according to the manufacturer’s instructions. The cDNA was used as a template in standard PCR reactions using GoTaq DNA Polymerase (Promega) and the following primers to amplify two regions in the *Poc1a* cDNA:

Exon 5–10 Fwd: 5’-CAAGACCAGCCGGGAATGTATC-3’
Exon 5–10 Rev: CTGGGGCTCGCCTTGAACTGACTC-3’
Optimal annealing temperature: 60°C.
Exon 7–8 Fwd: 5’- CCATCGGGAAACTACCTCATCAC-3’
Exon 7–8 Rev: 5’-AAATACTCCCCCGTTCTTG-3’

Optimal annealing temperature: 55°C. Sequencing was completed by the University of Michigan DNA Sequencing Core.

### Real-time quantitative PCR

TaqMan Universal PCR Master Mix (Applied Biosystems/Life Technologies) was used according to the manufacturer’s instructions. The following TaqMan probes were included to test expression levels of *Poc1a*: Exon 2–3 Assay ID: Mm01235877_m1. Exon 10–11 Assay ID: Mm01235875_m1. Reactions were loaded into MicroAmp Optical 96-well reaction plates (Applied Biosystems/Life Technologies) and run using an Applied Biosystems 7500 Real-Time PCR System.

### POC1A western blot analysis

Isolation of protein from postnatal day 3 tibiae and western blot analyses were carried out as previously described [75]. The blot was incubated with a 1:500 dilution of a mouse anti-human POC1A primary antibody (Abcam, ab67698) overnight at 4°C. The blot was incubated with a 1:5000 dilution of a goat anti-mouse IgG secondary antibody (Jackson ImmunoResearch Laboratories, Inc. #115-035-003) for one hour at room temperature. The blot was stripped and incubated with a 1:5000 dilution of a rat anti-yeast tubulin antibody (Abcam, ab6160) overnight at 4°C. The blot was incubated with a 1:10,000 dilution of a goat anti-Rat IgG secondary antibody (Jackson ImmunoResearch Laboratories, # 112-035-102) for one hour at room temperature. All antibodies were diluted in a blocking solution made up of 1% weight:volume Bovine Serum Albumin;Tris-buffered saline with Tween-20.

### Bone preparations and analysis

Adult skulls and were completely stripped of flesh by incubation in a dermastid beetle colony (http://www.lsa.umich.edu/ummz/mammals/dermestarium/default.asp.) Briefly, mice were euthanized, and bony parts were trimmed of excess flesh. These specimens were fed to the beetles. After the remaining soft tissues were removed, the bones were frozen at ~-20 C for 72 hrs, and the dead beetles removed.

To assess shape and mineralization of bones, mice were euthanized, bones were dissected and skin and flesh trimmed. Specimens were imaged in water using a cone beam microCT system (eXplore Locus SP; E Healthcare Pre-Clinical Imaging, London, ON, Canada). Scan parameters were 0.5 degree rotational increment, 4 frames averaged, 80 kVp/80 μA X-ray, and 0.508 mm Al filter plus beam flattener to reduce beam hardening artifacts [76]. Volumes were reconstructed at 18 μm isotropic voxel size and calibrated for grayscale value by a manufacturer-provided phantom of air, water, and hydroxyapatite-mimicking material.

### Bone and testis histology

Tibiae were dissected from 15 day old animals, fixed overnight in 4% paraformaldehyde (PFA), rinsed in PBS, and decalcified in 14% EDTA solution (weight:volume) for approximately 7 days, changing the solution each day. Testes were removed from mice of the listed ages and fixed overnight in Bouin’s Fixative Solution (Sigma). Both the testes and tibiae were then dehydrated through an ethanol series and embedded in paraffin. Sections were stained with hematoxylin and eosin (tibiae) or periodic acid-Schiff’s reagent (testes) according to standard protocols.

### Immunohistochemistry

Immunohistochemistry was conducted after removing paraffin from the samples by incubation of slides in xylenes and rehydration in an ethanol series to 1X PBS. Afterward, the testis sections were boiled in a 100 mM solution of citric acid (pH 6.0) for 10 minutes to expose epitopes, and cooled. Tibia sections were fixed for 10 minutes in 4% PFA, washed in PBS, and were either incubated with proteinase K in a buffer containing 50 mM Tris base, 1 mM EDTA and 0.5% Triton-X100 (acetylated tubulin), or placed in citric acid (pH 6.0) heated to 65°C for 1 hour (GM130, POC1A). Both testis and tibia sections were incubated for 20 minutes in solution of 3% hydrogen peroxide diluted in methanol to quench endogenous peroxidase activity. The slides were blocked in a solution included in the Tyramide Signal Amplification (TSA) Kit (Perkin-Elmer #SAT701001EA) or Mouse-On-Mouse (M.O.M) Blocking Reagent (Vector #BMK-2202) for one hour at room temperature. This was followed by an overnight incubation with primary antibodies in either TSA Block or Vector Mouse-On-Mouse Diluant at 4C. The rabbit anti-rat POC1A antibody was raised against rat POC1A aa 242–291, which is homologous to mouse POC1A aa 280–329. The mouse aa 269–308 comprise the WD7 repeat. The primary antibodies include: rabbit anti-rat POC1A (Abcam, ab135361, diluted 1:100), rabbit anti-human PLZF (Santa Cruz Biotechnology Inc., sc-22839 diluted 1:50), rabbit anti-SOX9 (Millipore, # AB5535, diluted 1:200), mouse anti-acetylated tubulin (Sigma #T6793, diluted 1:500), mouse anti-GM130 (BD Biosciences #610822, diluted 1:200), rabbit anti-KI67 (Novocastra NCL-Ki67p, diluted 1:250), rabbit anti-γH2AX (Ser139) (20E3) (Cell Signaling Technology #9718, diluted 1:500), and rabbit anti-c-KIT antibody (Cell Signaling Technology, # 3074S, diluted 1:400). Sections were washed in PBS, and incubated with the following secondary antibodies at room temperature: goat anti-rabbit biotin-conjugated secondary (Jackson ImmunoResearch Laboratories Inc., #111-067-003, POC1A, PLZF, SOX9, Ki67, γH2AX, and c-KIT for 1 hour), or the biotinylated anti-mouse secondary included in the M.O.M Kit from Vector Laboratories according to the manufacturer’s instructions. The subsequent steps were carried out according to the instructions provided in the TSA Fluorescein Tyramide Kit (TSA-FITC Kit) by Perkin-Elmer. Sections were counterstained with DAPI to reveal nuclei, cover slipped and photographed with a Leica Leitz DMRB/E compound microscope. Quantitation was done on three animals per genotype and age on 5–10 sections per individual and presented ± std. dev.

### MEF analysis

Mouse embryonic fibroblasts (MEFs) were isolated from individual embryos at embryonic day 13.5 by the University of Michigan Transgenic Animal Model Core, and stocks were genotyped for *Poc1a* ^cha/cha^ and frozen. MEFs were thawed, grown on gelatin-coated coverslips for two days, then serum treated or serum starved for 24 hours and fixed for 20 minutes in 4% paraformaldehyde at room temperature. The coverslips were washed in PBS, permeabilized in 0.1% SDS for 10 minutes at room temperature, and were incubated with the same primary antibodies for acetylated tubulin and GM130 listed above. The coverslips treated with acetylated tubulin primary antibody were then incubated in an Alexa 488 conjugated anti- mouse secondary (Life Technologies, # A-21141). The coverslips incubated with the GM130 primary were incubated with the M.O.M. anti-mouse Biotinylated IgG included in the M.O.M. Kit (Vector Laboratories). The GM130-incubated coverslips were then treated in the same way as the tissue sections according to the TSA-FITC Kit (Perkin-Elmer). All coverslips were counterstained with DAPI and photographed using an Olympus FluoView Laser Scanning Confocal Microscope (Microscopy and Image Analysis Laboratory, University of Michigan, Ann Arbor, MI).

### Apoptosis analysis by TUNEL staining

Sections through P15 tibiae were de-waxed in xylene, rehydrated through an ethanol series to 1 X PBS, and then pretreated by incubating the slides in citric acid (pH 6.0) at 65°C for 1 hour. Slides were washed in PBS and treated according to the manufacturer’s instructions thereafter (Roche *In Situ* Cell Death Detection Kit, Fluorescein, #11684795910). Quantification was done on three separate sections from two different mice of each genotype and presented as average per section ± standard error. The two-tailed T test was applied to assess significance.

### Spermatogonial transplantation assays

Rosa-Lac Z mice (Jackson Laboratories, Bar Harbor, ME, #002073) were crossed to *Poc1a* ^cha/cha^ homozygous females to generate reporter positive heterozygous mice for intercrossing and generation of reporter positive wild type and mutant males for the experiments requiring labeled mutant germ cells. Spermatogonial stem cell transplantation was performed as described previously [77]. Briefly, single cell suspensions from donor testes were generated by two-step enzymatic digestion and spermatogonia were enriched by selection with a 30% continuous Percoll gradient. Adult wild type recipient mice were prepared by busulfan treatment (60 mg/kg of body weight) at least 6 weeks before transplantation to deplete endogenous germ cells as described previously [77]. Adult *Poc1a* mutant recipient mice were not prepared with busulfan treatment because the endogenous germline was already depleted. For experiments involving *Poc1a* mutant and wild type counterparts as SSC donors, NCr nude mice (Taconic) were used as recipients to avoid immunological incompatibility. For all transplantations, donor cells were re-suspended in mouse serum-free injection media [78] at 1X10^6^ cells/ml and approximately 10 μl of cell suspension was microinjected into the seminiferous tubules of each recipient testis. The recipient testes were examined for donor-derived colonies of spermatogenesis via X-Gal staining ~3 months after transplantation.

### Protein structure prediction

The molecular structure of mouse POC1A was predicted using the WD40 structure predictor algorithm (WDSP) [34] and models generated using PyMOL (The PyMOL Molecular Graphics System, Version 1.7.2.2 Schrödinger, LLC).

## Supporting Information

S1 Table Intronic sequence variants detected in *chagun mutant* by exome sequence analysis. (DOCX)

S2 Table Additional genes on RP24-384G5 and allele phenotypes. (DOCX)

S1 Fig Insertion of *Cenpw* cDNA into *Poc1a* exon 8. Panel A. The Genome Browser view reveals mismatched paired end sequences and low coverage near *Poc1a* exon 8. Screenshots from the Broad Institute’s Integrative Genomics Viewer (IGV) show a drop in paired end sequence coverage within exon 8 of *Poc1a*. Pink reads indicate that the other end of the mate pair maps a different region of the genome. The location of exon 8 is indicated by the red rectangles under the sequence reads. (CVG: coverage) B. DNA sequences relevant to the *chagun* mutation. *Poc1a* wild type exon 8: The genomic sequence of exon 8 of wild type mouse *Poc1a* is highlighted in blue, and the insertion site is indicated by an arrow. The LINE-1 target site duplication is underlined. *Cenpw* cDNA: The DNA sequence of the longest *Cenpw* cDNA is indicated, and the portion of it that is inserted into *Poc1a* in the *chagun* mutants is indicated in grey. Seven bp at the 5’ end of the *Cenpw* transcript are missing in the *Poc1a* insertion. Multiple *Cenpw* ESTs with a variety of polyadenylation sites have been reported. The polyadenylation site used in the cDNA inserted into *Poc1a* is 24 bp downstream from ATTAAA (AUUAAA), which is the second most common polyadenylation signal sequence in animals after AATAAA (AAUAAA). This suggests that a nearly complete primary transcript of *Cenpw* could have been reverse transcribed and inserted into *Poc1a*. *Poc1a chagun* exon 8: The reverse complement of a portion of the *Cenpw* cDNA (in grey) is inserted into exon 8 of *Poc1a* (blue) to create the *chagun* mutation, which also includes an insertion of 61 thymidines (highlighted in yellow), and a target site duplication. The primers used to detect splicing into exon 8 were: Forward: 5’-CCATCGGGAAACTACCTCATCAC-3’ and Reverse: 5’-AAATACTCCCCCGTTCTTG-3’. The ΔΔC_T_ values for the qRT-PCR using a Taqman probe 5’ to the *Cenpw* insertion relative to *Gapdh* were 0.1775 (0.8842 of wild type) and the probe 3’ to the *Cenpw* insertion relative to *Gapdh* was 0.2004 (0.8702 of wild type).
(TIF)

S2 Fig Structure of *Poc1a* mutant allele. The *Poc1a* ^tm1(KOMP)Mbp^ mutant allele generated by the KOMP deletes exons three through 6, and replaces them with a lacZ expression cassette that will be driven by the *Poc1a* promoter. It includes a splice acceptor (En2 SA), an internal ribosome entry site (IRES), and sequences that regulate termination and polyadenylation (pA). The insertion also includes a selection cassette that confers neomycin resistance (neo). The selection portion can be removed with *cre*-mediated excision at the loxP sites, and the entire cassette can be deleted with *flp*-mediated excision at the FRT sites. If splicing occurs between exon 2 and exon 7, the protein will be in frame, but the fifth and a portion of the sixth of the seven WD40 repeat domains would be missing. The *Poc1a* ^cha^ mutant allele causes skipping of exon 8, and the predicted protein is in frame but lacking a portion of the seventh WD40 repeat domain. The *Cenpw* cDNA insertion is indicated by the black box.
(DOCX)

S3 Fig POC1A is expressed in the germ cells and Sertoli cells. POC1A immunostaining (green) was carried out on testis sections from normal and *Poc1a* ^tm1(KOMP)Mbp^ (*Poc1a* ^-/-^) mutant mice collected at postnatal day 21 and 40. Scale bars as indicated.
(TIF)

S4 Fig Chondrocyte abnormalities in the growth plate of of the *Poc1a* ^cha/cha^ mutants. Hematoxylin and eosin staining was performed on sections of the proximal tibia of wild type and *Poc1a* ^cha/cha^ mutants. The *Poc1a* ^cha/cha^ tibia growth plate lacks the columnar cellular organization observed in the wild type tibial growth plate. Scale bar = 100 μm.
(TIF)

S5 Fig γH2AX expression in *Poc1a* ^cha/cha^ testes at P7 and P14. Immunohistochemistry for γH2AX was performed on testis sections from P7 and P14 wild type and *Poc1a* ^cha/cha^ males. The stained sections at P7 were used for the quantification shown in Fig 8D. Scale bar = 100μm.
(TIF)

S6 Fig PLZF expression in testis of young wild type and mutant mice. Sections from wild type and *Poc1a* ^cha/cha^ mouse testes collected from P7 and P14 mice were immunostained with antibodies specific for PLZF and counterstained with DAPI. Scale bar = 100μm.
(TIF)

S7 Fig *Poc1b* is expressed in testis and bone. RT-PCR analysis of *Poc1b* expression was carried out with cDNA from testis of normal mice at P21 and from tibia of normal mice at P3 and 3 wk (top panel). Genomic DNA and no template controls were included. The same reactions were carried out on RNA samples with no reverse transcriptase (bottom panel).
(TIF)
