# Supplementary material for: LINE-1 Mediated Insertion into Poc1a (Protein of Centriole 1 A) Causes Growth Insufficiency and Male Infertility in Mice
Source: PLoS Genet. 2015 Oct 23;11(10):e1005569. doi: 10.1371/journal.pgen.1005569 (PMC4619696; doi:10.1371/journal.pgen.1005569)
Supplement: S1 Table — (DOCX) [file pgen.1005569.s001.docx]

| **S1 Table. Intronic sequence variants detected in *chagun* mutant by exome sequence analysis** | | | | | |
| --- | --- | --- | --- | --- | --- |
| **Gene Name** | **Gene Name and Function** | **Coverage** | **Type of Variant** | **Variant Location (Chr9, Mm9)** | **Conclusions** |
| ***Ephb1*** | Eph receptor B1 | 64X | Insertion (N/A) | 101844999 | Gene sequenced by Sanger Method—No variants noted |
| ***Cpne4*** | Copine IV | 6X | Insertion (+TT) | 104588727 | In Poly-T Tract |
| ***Atp2c1*** | ATPase, Ca+2 sequestering | 57X | SNP (A🡪G) | 105337374 | Not in splice site or branch point |
| ***Atp2c1*** | ATPase, Ca+2 sequestering | 59X | Deletion (-1) | 105337375 | Not in splice site or branch point |
| ***Pik3r4*** | Phosphatidylinositol-3-kinase, regulatory subunit, polypeptide 4, p150 | 42X | Deletion (-A) | 105547333 | In Poly-T tract |
| ***Col6a6*** | Collagen, type VI, alpha 6 | 7X | SNP (C🡪T) | 105625146 | In a CA-repeat |
| ***Col6a6*** | Collagen, type VI, alpha 6 | 8X | Insertion (+G) | 105641565 | Not in a splice site or branch point |
| ***Col6a4*** | Collagen, type VI, alpha 4 | 9X | Deletion (-G) | 105969540 | In Poly-G Tract |
| ***Col6a4*** | Collagen, type VI, alpha 4 | 8X | SNP (C🡪T) | 105969546 | In Poly-G Tract |
| ***Alas1*** | Aminolevulinic acid synthase 1 | 48X | SNP (T🡪G) | 106136553 | In Poly-T Tract |
| ***Acy1*** | Aminoacylase 1 | 170X | SNP (A🡪G) | 106337184 | Not in splice site or branch point, not well conserved |
| ***Parp3*** | Poly (ADP-ribose) polymerase family, member 3 | 2X | SNP (A🡪G) | 106377303 | Not conserved, in poly-G tract |
| ***Parp3*** | Poly (ADP-ribose) polymerase family, member 3 | 2X | SNP (A🡪G) | 106377304 | Not Conserved, in poly-G tract |

*A: Adenosine; T:Thymosine; C: Cytosine; G: Guanosine

**SNP: Single Nucleotide Polymorphism

***Chr9: Mouse Chromosome 9

****Mm9: *Mus musculus* genome sequence, build 9
