# Supplementary material for: LINE-1 Mediated Insertion into Poc1a (Protein of Centriole 1 A) Causes Growth Insufficiency and Male Infertility in Mice
Source: PLoS Genet. 2015 Oct 23;11(10):e1005569. doi: 10.1371/journal.pgen.1005569 (PMC4619696; doi:10.1371/journal.pgen.1005569)
Supplement: S2 Table — (DOCX) [file pgen.1005569.s002.docx]

| **S2 Table. Additional Genes on RP24-384G5 and Allele Phenotypes** | | | |
| --- | --- | --- | --- |
| **Gene Name** | **Allele** | **Allele Phenotype** | **Expression** |
| *Twf2* | Yes  Insertion of a neo cassette into exon 1 | No phenotype reported.  Homozygotes are viable and fertile.  129 background strain | Limb bud  Mandible and maxilla  Spermatocytes  Spermatogenic cells |
| *Tlr9* | Yes  Several chemically induced mutations and a single knock-out allele | All alleles report impaired immunity and susceptibility to specific pathogens | Testis |
| *Alas1* | Yes  A targeted GFP reporter | Homozygotes for the reporter allele exhibit early embryonic lethality.  No strain data available | Limb bud  Mandible and maxilla  Osteoblast  Spermatogenic cells  Testis |
| *Dusp7* | No  A targeted knock out and a LacZ reporter allele exist for related *Dusp6* | Dusp6 causes a skeletal growth defect that is highly variable but evident in heterozygotes | Limb bud  Mandible and maxilla |

All information compiled from the Mouse Genome Informatics Website (The Jackson Laboratory, Bar Harbor, Maine)
