# Supplementary figures and images for: LINE-1 Mediated Insertion into Poc1a (Protein of Centriole 1 A) Causes Growth Insufficiency and Male Infertility in Mice
Source: PLoS Genet. 2015 Oct 23;11(10):e1005569. doi: 10.1371/journal.pgen.1005569 (PMC4619696; doi:10.1371/journal.pgen.1005569)

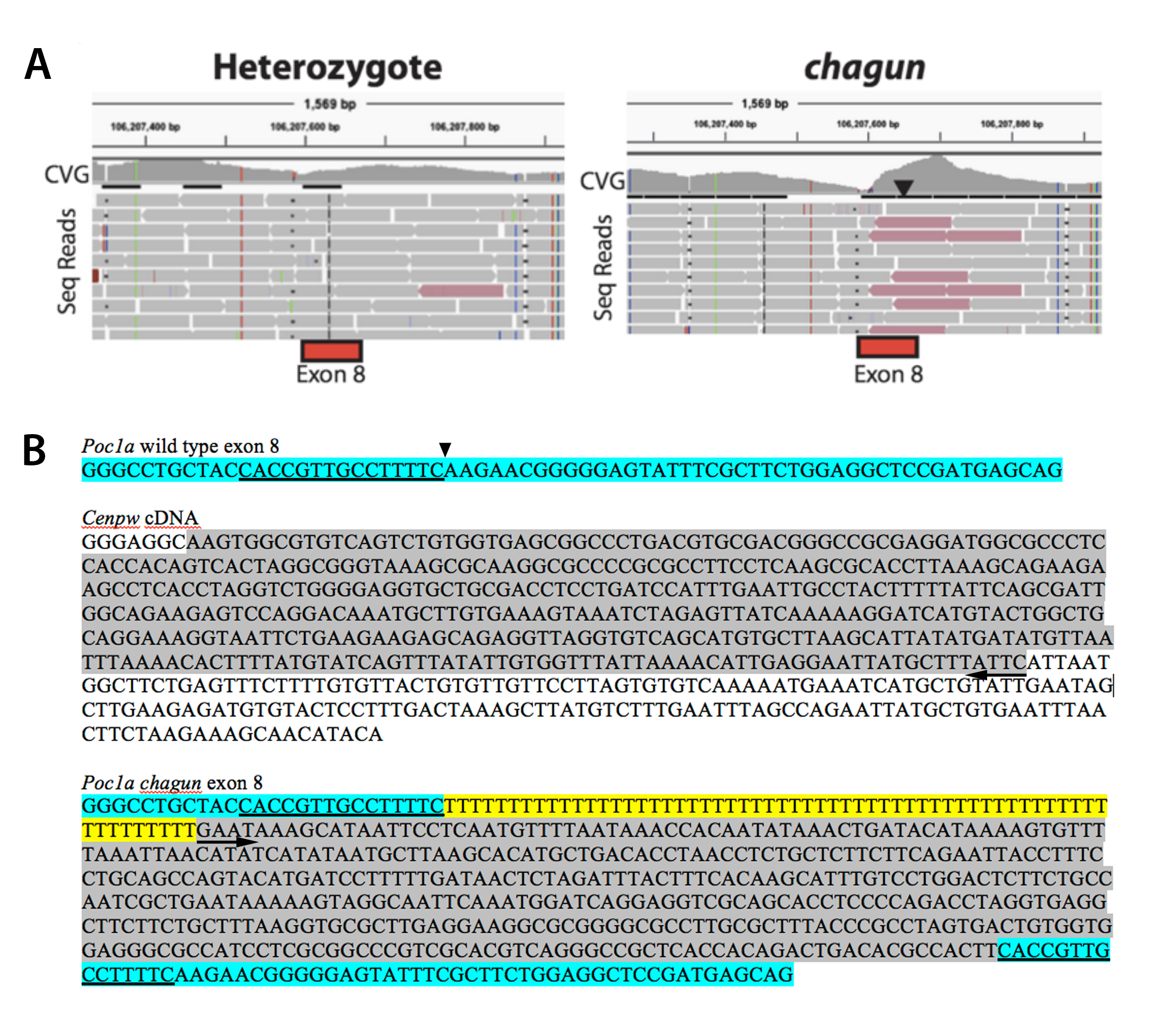

Supplement: S1 Fig — Panel A. The Genome Browser view reveals mismatched paired end sequences and low coverage near Poc1a exon 8. Screenshots from the Broad Institute’s Integrative Genomics Viewer (IGV) show a drop in paired end sequence coverage within exon 8 of Poc1a. Pink reads indicate that the other end of the mate pair maps a different region of the genome. The location of exon 8 is indicated by the red rectangles under the sequence reads. (CVG: coverage) B. DNA sequences relevant to the chagun mutation. Poc1a wild type exon 8: The genomic sequence of exon 8 of wild type mouse Poc1a is highlighted in blue, and the insertion site is indicated by an arrow. The LINE-1 target site duplication is underlined. Cenpw cDNA: The DNA sequence of the longest Cenpw cDNA is indicated, and the portion of it that is inserted into Poc1a in the chagun mutants is indicated in grey. Seven bp at the 5’ end of the Cenpw transcript are missing in the Poc1a insertion. Multiple Cenpw ESTs with a variety of polyadenylation sites have been reported. The polyadenylation site used in the cDNA inserted into Poc1a is 24 bp downstream from ATTAAA (AUUAAA), which is the second most common polyadenylation signal sequence in animals after AATAAA (AAUAAA). This suggests that a nearly complete primary transcript of Cenpw could have been reverse transcribed and inserted into Poc1a. Poc1a chagun exon 8: The reverse complement of a portion of the Cenpw cDNA (in grey) is inserted into exon 8 of Poc1a (blue) to create the chagun mutation, which also includes an insertion of 61 thymidines (highlighted in yellow), and a target site duplication. The primers used to detect splicing into exon 8 were: Forward: 5’-CCATCGGGAAACTACCTCATCAC-3’ and Reverse: 5’-AAATACTCCCCCGTTCTTG-3’. The ΔΔCT values for the qRT-PCR using a Taqman probe 5’ to the Cenpw insertion relative to Gapdh were 0.1775 (0.8842 of wild type) and the probe 3’ to the Cenpw insertion relative to Gapdh was 0.2004 (0.8702 of wild type). (TIF) [file pgen.1005569.s003.tif]

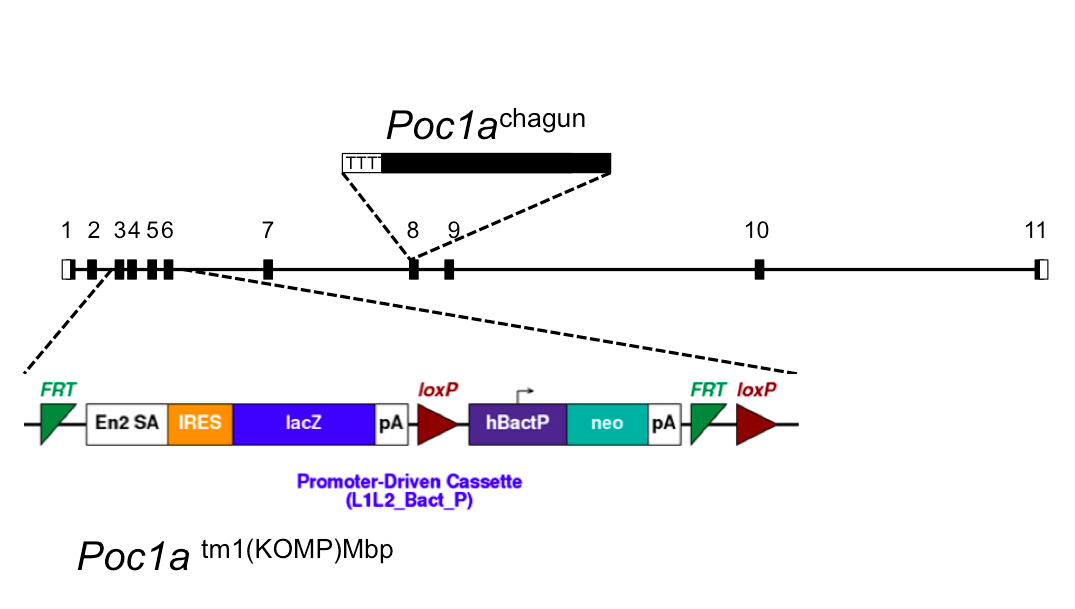

Supplement: S2 Fig — The Poc1a tm1(KOMP)Mbp mutant allele generated by the KOMP deletes exons three through 6, and replaces them with a lacZ expression cassette that will be driven by the Poc1a promoter. It includes a splice acceptor (En2 SA), an internal ribosome entry site (IRES), and sequences that regulate termination and polyadenylation (pA). The insertion also includes a selection cassette that confers neomycin resistance (neo). The selection portion can be removed with cre-mediated excision at the loxP sites, and the entire cassette can be deleted with flp-mediated excision at the FRT sites. If splicing occurs between exon 2 and exon 7, the protein will be in frame, but the fifth and a portion of the sixth of the seven WD40 repeat domains would be missing. The Poc1a cha mutant allele causes skipping of exon 8, and the predicted protein is in frame but lacking a portion of the seventh WD40 repeat domain. The Cenpw cDNA insertion is indicated by the black box. (DOCX) [file pgen.1005569.s004.docx]

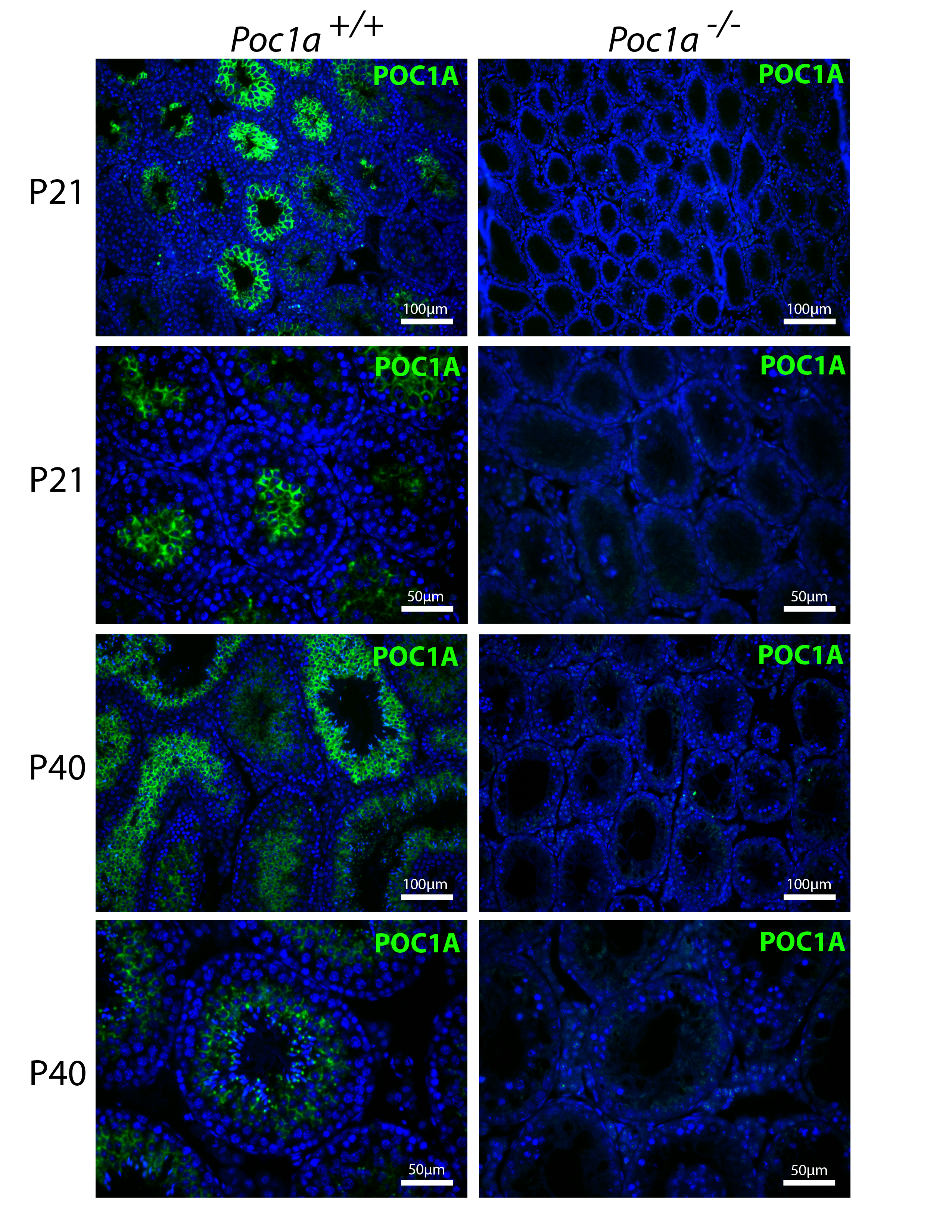

Supplement: S3 Fig — POC1A immunostaining (green) was carried out on testis sections from normal and Poc1a tm1(KOMP)Mbp (Poc1a -/-) mutant mice collected at postnatal day 21 and 40. Scale bars as indicated. (TIF) [file pgen.1005569.s005.tif]

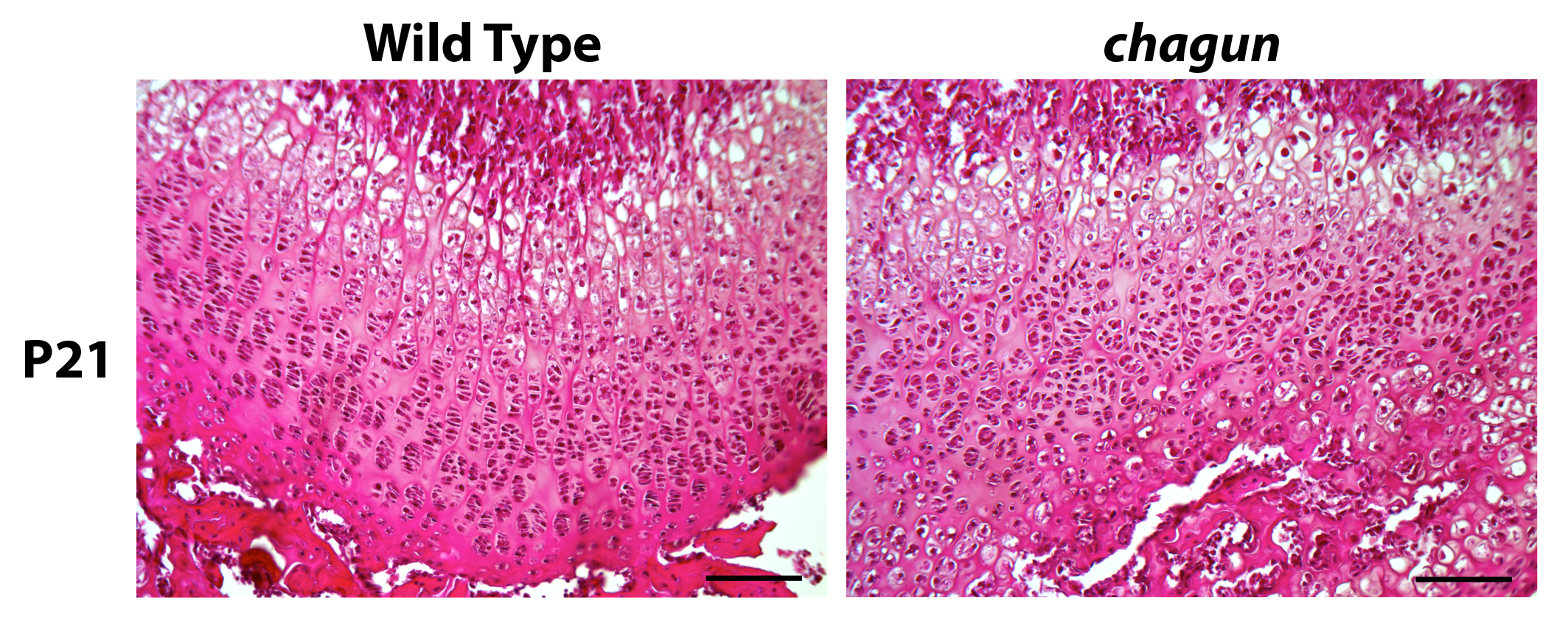

Supplement: S4 Fig — Hematoxylin and eosin staining was performed on sections of the proximal tibia of wild type and Poc1a cha/cha mutants. The Poc1a cha/cha tibia growth plate lacks the columnar cellular organization observed in the wild type tibial growth plate. Scale bar = 100 μm. (TIF) [file pgen.1005569.s006.tif]

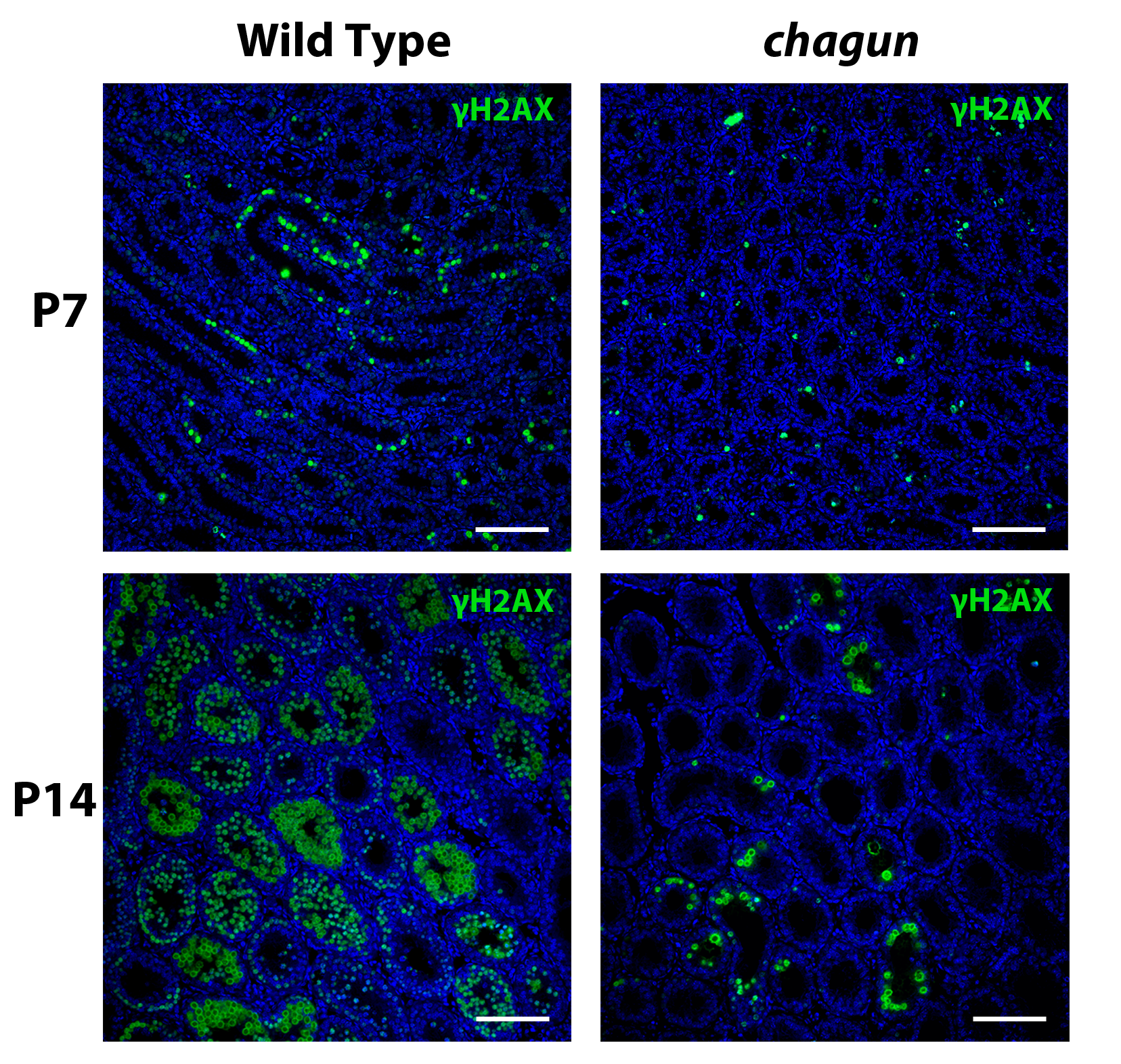

Supplement: S5 Fig — Immunohistochemistry for γH2AX was performed on testis sections from P7 and P14 wild type and Poc1a cha/cha males. The stained sections at P7 were used for the quantification shown in Fig 8D. Scale bar = 100μm. (TIF) [file pgen.1005569.s007.tif]

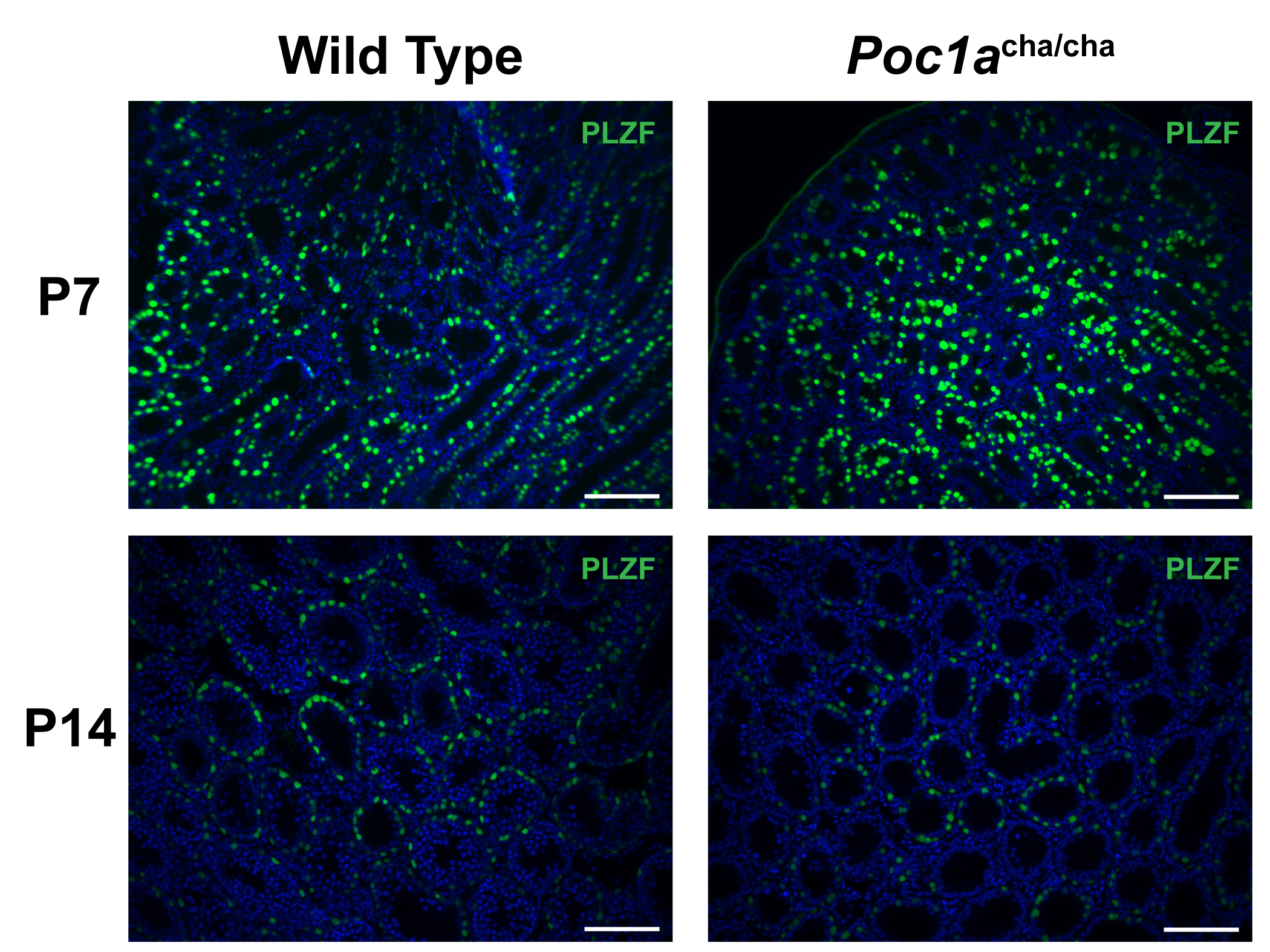

Supplement: S6 Fig — Sections from wild type and Poc1a cha/cha mouse testes collected from P7 and P14 mice were immunostained with antibodies specific for PLZF and counterstained with DAPI. Scale bar = 100μm. (TIF) [file pgen.1005569.s008.tif]

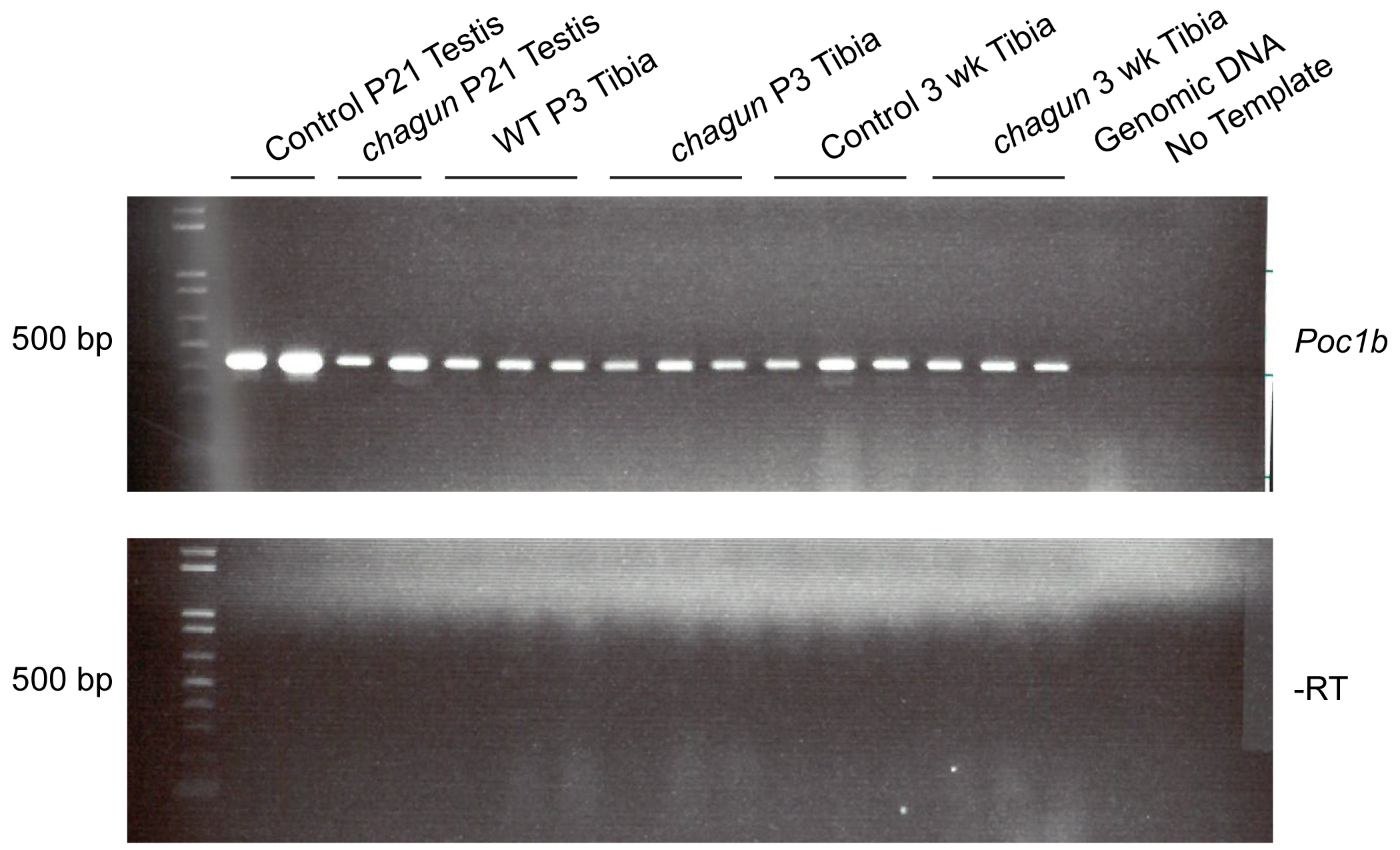

Supplement: S7 Fig — RT-PCR analysis of Poc1b expression was carried out with cDNA from testis of normal mice at P21 and from tibia of normal mice at P3 and 3 wk (top panel). Genomic DNA and no template controls were included. The same reactions were carried out on RNA samples with no reverse transcriptase (bottom panel). (TIF) [file pgen.1005569.s009.tif]
